# Unveiling an unexpected superoxide-mediated photooxidation mechanism of squalene monohydroperoxides to squalene hydroperoxy cyclic peroxides through ESR and LC–MS/MS analyses

**DOI:** 10.1038/s41598-023-46044-9

**Published:** 2023-11-09

**Authors:** Saoussane Khalifa, Masaru Enomoto, Kiyotaka Nakagawa

**Affiliations:** 1 Laboratory of Food Function Analysis, Graduate School of Agricultural Science, Tohoku University, Sendai, 980-8572 Japan; 2 Applied Bioorganic Chemistry Laboratory, Graduate School of Agricultural Science, Tohoku University, Sendai, 980-8572 Japan

**Keywords:** Chemical biology, Chemistry

## Abstract

Lipid cyclic peroxides are a rarely reported and documented class of compounds in the human organism. Recently, we reported the formation of squalene (SQ) hydroperoxy cyclic peroxides derived from SQ monohydroperoxide isomers (SQ-OOHs) for the first time. Notably, we successfully detected and quantified cis-2-OOH-3-(1,2-dioxane)-SQ in the human skin. Nevertheless, the underlying mechanism governing the formation of these compounds remained elusive. Therefore, in the current study, we set to determine the reaction’s mechanism. To this end, a comprehensive analysis of the precise conditions involved in the onset and propagation of this conversion was carried out by oxidizing total SQ-OOHs under different conditions, including singlet oxygen (^1^O_2_), thermal, and photoinduced oxidations monitored by quantifying the generated 2-OOH-3-(1,2-dioxane)-SQ using liquid chromatography-tandem mass spectrometry (LC–MS/MS). Radical intermediates were thoroughly investigated using Electron Spin Resonance (ESR) with the aid of spin traps and radical references. Moreover, calculations of SQ-OOHs’ electrostatic charges were performed on Spartan 18 software. We found that the reaction is ideally induced and favored under photooxidation in the presence of ^3^O_2_ in hexane, and that superoxide radical (O_2_^•−^) is the first key intermediate in this mechanism, whereas peroxyl radicals were the major species observed throughout the oxidation. Chemical calculations provided an explanation for the targeting of tertiary SQ-OOHs by this reaction and gave further evidence on the proposed heterolytic cleavage initiating the reaction. The novel oxidation mechanism suggested herein offers new insights into understanding lipid secondary oxidation and is a promising finding for further studying lipid cyclic peroxides in general.

## Introduction

Modifications and rearrangements of organic peroxides have been the subject of numerous studies over the years, marked by the breakthrough discovery of some of the early in vivo organic endoperoxide intermediates, products of the modification of arachidonic acid hydroperoxides. These intermediates serve as precursors to the bioactive hormonal class recognized today as prostaglandins, pioneering studies that were conducted by Samuelsson B.I., Bergström S. K., and Vane J. R., which yielded them the Nobel Prize in 1982^1,2^. Interest in organic cyclic and endoperoxides grew over the years, to include the therapeutically bioactive artemisinin discovered in the ’70s by Tu Youyou^3^, and its derivatives reported in the following decades, proven as effective antimalarial agents^3^. Similarly, 1,2-dioxane containing compounds isolated from marine species such as Plakortin and its derivatives have been reported to possess antimalarial activities^4–7^, giving further importance to investigate novel organic cyclic peroxides, their mechanisms of formation, and roles in the organism. Later reports by Frankel et al. and other authors^8–16^ targeted lipid cyclic peroxides, where they demonstrated the formation of hydroperoxy cyclic peroxides from methyl linoleate and methyl linolenate in vitro. Their findings point out the formation of both 1,2-dioxolane and 1,2-dioxane hydroperoxyl oxidation products, as well as the proposition of several hypotheses regarding the oxidation mechanism involved in their formation. A drawback in the advances to understand this phenomenon is the lack of attempts and evidence to fully understand the mechanism by which these cyclic peroxides are formed from lipid hydroperoxides (LOOHs). The aforementioned studies have primarily postulated the initiation of the mechanism by the formation of a peroxyl radical from a firstly formed hydroperoxide, subsequently leading to a chain reaction. Nevertheless, corroborating evidence supporting the formation of peroxyl radicals from LOOHs could not be found. Given the presumably low O–O bond dissociation energy (BDE) compared to that of O–H, solid proof is needed to confirm the proposed pathway.

While multiple mechanisms involved in organic and lipid hydroperoxides’ modifications ranging from the well documented Baeyer − Villiger oxidation, Russel’s lipid hydroperoxides’ self-reaction, hock fragmentation, and the Criegee mechanism, to the lesser known ones such as the Kornblum − DeLaMare rearrangement, Schenck and Smith rearrangements^17,18^, have all been thoroughly investigated, confirmed, and have been successfully employed as useful tools in organic synthesis. Attempts to elucidate and understand the mechanism leading to the formation and decomposition of lipid hydroperoxy cyclic peroxides from LOOHs remain scarce. Moreover, the eventual final products of lipid oxidation are commonly believed to be decomposition or breakdown products, leading to the formation of volatile aldehydes and ketones. These compounds have long been considered as oxidative stress biomarkers. The most prominent of which, 4-hydroxynonenal (HNE) and malondialdehyde (MDA)^19,20^, have been reported to originate from lipid hydroperoxy cyclic peroxide intermediates^19^. This further highlights the importance and role of cyclic peroxides in lipid oxidation in general. Therefore, understanding their mechanism of formation from LOOHs is deemed crucial.

**Figure 1:**
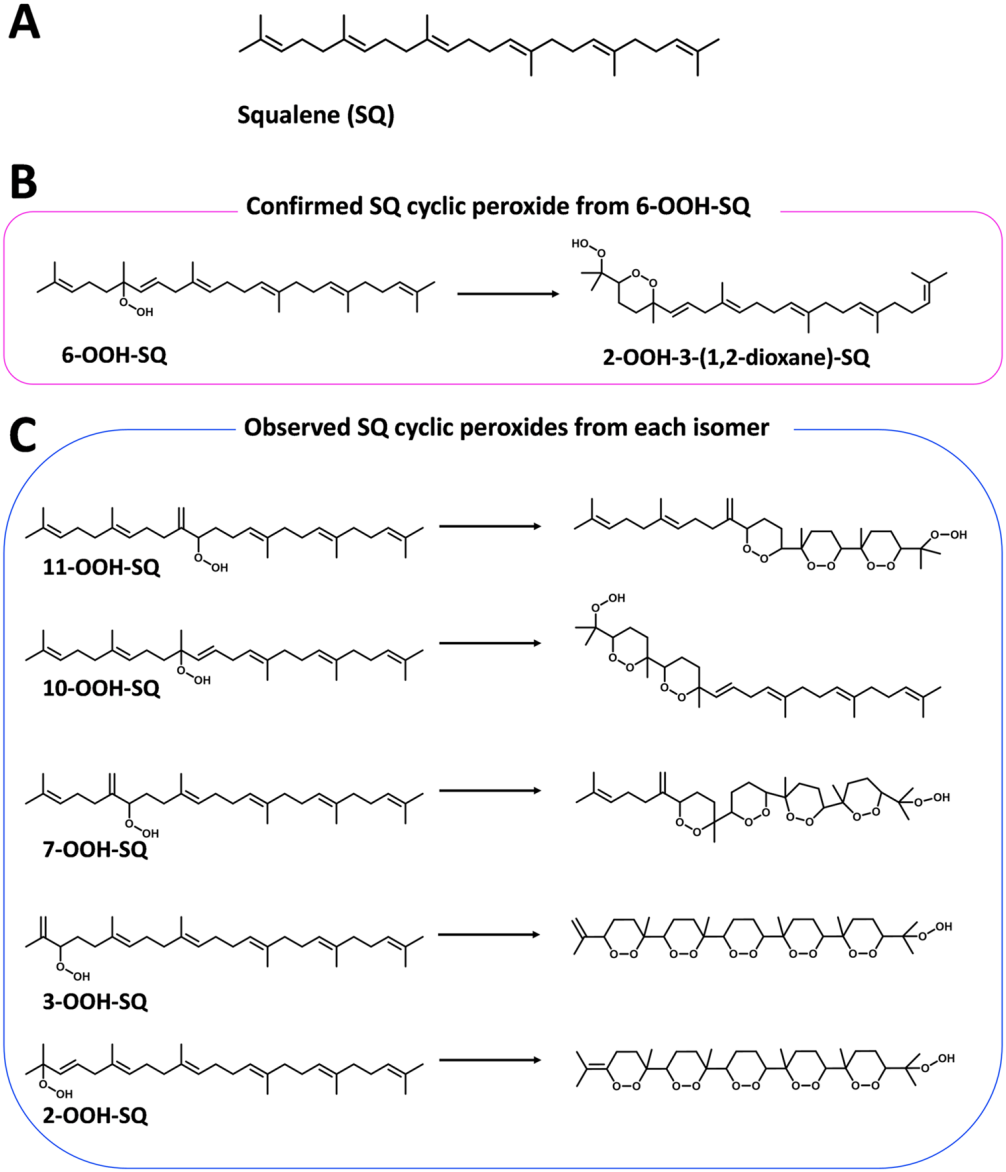
Chemical structures of (**A**) SQ, (**B**) 6-OOH-SQ and 2-OOH-3-(1,2-dioxane)-SQ, and (**C**) SQ-OOHs and their respective hydroperoxy cyclic peroxides.

Squalene (SQ) (Fig. 1A) is an endogenous triterpenoid of the human organism and many other living species. We recently reported for the first time the formation of SQ hydroperoxy cyclic peroxides (Fig. 1B,C) derived from SQ monohydroperoxides (SQ-OOHs), as well as the detection and quantification of 2-OOH-3-(1,2-dioxane)-SQ (Fig. 1B), product of 6-OOH-SQ, in human skin surface lipids^21^. Although the role and function of 2-OOH-3(1,2-dioxane)-SQ in the skin are not fully understood yet, it is of utmost importance to elucidate the underlying mechanism governing the formation of SQ hydroperoxy cyclic peroxides. This pursuit is crucial as it holds the potential to provide broader insights into the formation of cyclic peroxides from LOOHs in general, and to help understand the role and effect of these compounds in the organism. To this end, in the herein study, we investigated the mechanism involved in the formation of SQ hydroperoxy cyclic peroxides by, first, determining the exact conditions leading to and favoring their formation by monitoring the generation of 2-OOH-3-(1,2-dioxane)-SQ from SQ-OOHs using LC–MS/MS under photooxidation, singlet oxygen (^1^O_2_) oxidation, thermal oxidation in the presence and absence of a radical initiator, the effect of ozone which is in constant contact with the human skin was also examined. Next, we investigated the radical intermediate species formed during the reaction under optimal conditions using Electron Spin Resonance (ESR) and spin traps in the presence and absence of superoxide dismutase (SOD), and by using radical references for the identification of the species. Lastly, to help formulate our hypothesis, we conducted some simple chemical calculations on the electrostatic charges of the precursor compounds using Spartan 18 software to elucidate the targeting of tertiary SQ-OOHs compared to its secondary isomers. The obtained results show an unexpected novel superoxide (O_2_^•−^) mediated photooxidation mechanism that holds promising applications to further understand LOOHs oxidation in general.

## Materials and methods

### Chemicals

3-(1,4-Epidioxy-4-methyl-1,4-dihydro-1-naphthyl) propionic acid (EP) (≥ 90%) was obtained from WakenBtech Co., Ltd (Kyoto, Japan). 5,5-Dimethyl-1-pyrroline N-oxide (DMPO) (≥ 97%), potassium superoxide (KO_2_), 18-Crown-6 (≥ 98%), 2,2'-Azobis(4-methoxy-2,4-dimethylvaleronitrile) (MeO-AMVN) (≥ 95%), tert-butyl hydroperoxide (tBuOOH) solution (70%), hydrogen peroxide (H_2_O_2_) solution (30%), rose bengal (RB), and SOD from Bovine Erythrocytes (Cu/Zn Type) were purchased from FUJIFILM Wako Pure Chemical Industries, Ltd (Osaka, Japan). N-tert-Butyl-α-(4-pyridyl-1-oxide)nitrone (POBN) (≥ 98%) was obtained from Tokyo Chemical Industry Co., Ltd (Tokyo, Japan). All other reagents were of the highest grade available.

### Preparation of SQ-OOH isomers and 2-OOH-3-(1,2-dioxane)-SQ

SQ-OOH isomers (≥ 98%) and 2-OOH-3-(1,2-dioxane)-SQ (≥ 99%) were prepared according to our previously described methods^21^. In brief, SQ-OOHs were obtained by ene reaction of SQ following a photosensitized ^1^O_2_ (generated by a type II photosensitizer (RB)) oxidation mechanism. The generation of ^1^O_2_ was confirmed by its monomeric phosphorescence at 1270 nm on an FP 8700 NIR spectrofluorometer (Fig. S1, Electronic Supplementary Information (ESI)). The oxidation was followed by RB’s quenching and several purification procedures. 2-OOH-3-(1,2-dioxane)-SQ was prepared by exposing SQ-OOHs to photons in the presence of triplet state molecular oxygen (^3^O_2_) followed by several purifications, the precursor of 2-OOH-3-(1,2-dioxane)-SQ is known to be 6-OOH-SQ based on our previous study^21^.

### Determination of the optimal oxidative conditions involved in the formation of 2-OOH-3-(1,2-dioxane)-SQ: exposure of SQ-OOHs to different oxidative conditions

Previously, we reported that 6-OOH-SQ and 10-OOH-SQ were the main targets of the photooxidation of total SQ-OOHs isomers, resulting respectively in the rather stable 2-OOH-3-(1,2-dioxane)-SQ detected on the human skin, and the unstable 2-OOH-3,7-di(1,2-dioxane)-SQ^21^. We also demonstrated that the oxidation of individual SQ-OOHs resulted in similar SQ hydroperoxy cyclic peroxides, showing a repetitive pattern of a chain reaction mechanism occurring in all isomers^21^. However, the exact optimal conditions and parameters favoring and involved in the formation of these SQ hydroperoxy cyclic peroxides were not well understood nor thoroughly investigated.

To determine the oxidation mechanism underlying the formation of SQ hydroperoxy cyclic peroxides, and in particular, in the formation of 2-OOH-3-(1,2-dioxane)-SQ, first, a series of experiments under varied oxidative conditions was conducted to determine the precise factors involved in its formation. The reactions were monitored by measuring the amount of 2-OOH-3-(1,2-dioxane)-SQ formed over 8 h of SQ-OOHs’ oxidation by LC–MS/MS on a 4000 quadrupole/linear ion-trap tandem mass spectrometer (4000QTRAP) (SCIEX, Tokyo, Japan) using our recently reported method^21^. SQ-OOHs were oxidized under the following conditions:

#### Under ^1^O_2_ oxidation

To 0.5 µg/200 µL solution of SQ-OOHs in methanol/chloroform (1:1), 10 µL of an EP solution in methanol/chloroform (1:1) was added to reach a final concentration of 20 mM. The mixture was incubated at 25 °C, the reaction was carried out in triplicate and monitored over 8 h. Sampling was conducted at 0 h, 1 h, 2 h, 4 h, 6 h and 8 h. ^1^O_2_ generation from the decomposition of EP in the presence and absence of SQ-OOHs was measured by its monomeric phosphorescence at 1270 nm on an FP 8700 NIR spectrofluorometer from 4 mg of EP in deuterated methanol/chloroform (1:1) as the lifetime of ^1^O_2_ was shown to be longer in deuterated solvents^22^. The decomposition of EP in the presence of SQ-OOHs was further confirmed by LC-UV (mobile phase: methanol/water 90:10; column: COSMOSIL 5C18-MS-II (4.6 × 250 mm, NACALAI TESQUE, INC., Kyoto, Japan); flow rate: 1 mL/min; oven temperature: 40 °C; wavelength: 210 nm), Q1 scan in a 6500Qtrap (SCIEX, Tokyo, Japan), and ^1^H NMR (Varian NMR (Palo Alto, CA, USA) Unity 400-MRTT spectrometer at 400 MHz using tetramethylsilane (TMS) as the reference). The changes in SQ-OOHs were also verified by a LC-UV method reported in our previous study^21^.

#### Under thermal oxidation (presence and absence of a radical initiator)

Oxidation was carried out at 60 °C in the presence and absence of the radical initiator MeO-AMVN as follows: to 0.5 µg of pure SQ-OOHs in amber vials, MeO-AMVN (dissolved in chloroform) at a final quantity of 1 μg was added, after thorough vortexing, solvents were evaporated from the recipients. Samples with and without MeO-AMVN were purged with ^3^O_2_, enclosed with parafilm and incubated at 60 °C in an oil bath for 8 h. The reaction was carried out in triplicate and monitored over 8 h, sampling was conducted at 0 h, 1 h, 2 h, 4 h, 6 h and 8 h.

#### Exposure to photons and ^3^O_2_

The above conditions were compared to the conditions under which 2-OOH-3-(1,2-dioxane)-SQ was initially noticed to form, which are exposure of SQ-OOHs to photons in the presence of ^3^O_2_ in hexane. SQ-OOHs, 0.5 µg/200 µL hexane in transparent vials purged with ^3^O_2_, were exposed to an LED light of 60 Klux intensity. The reaction was maintained at 4 °C, performed in triplicate, and monitored over 8 h. Sampling was conducted at 0 h, 1 h, 2 h, 4 h, 6 h and 8 h.

In all of the above experiments, prior to LC–MS/MS analysis, the samples were evaporated then dissolved in 100% methanol. The concentrations of the formed 2-OOH-3-(1,2-dioxane)-SQ were determined by external standard curves using the pure synthesized standard of 2-OOH-3-(1,2-dioxane)-SQ. All samples were stored at − 80 °C and purged with nitrogen until analyses.

Apart from the above oxidative conditions, exposure of SQ to ozone, a prevalent air pollutant that interacts with skin surface lipids, was also examined to assess its effect on SQ and the possibility of its involvement in the formation of 2-OOH-3-(1,2-dioxane)-SQ previously detected on the skin. In brief, either SQ or SQ-OOHs at a concentration of 0.01% in isopropanol was exposed to O_3_ at a flow rate of 1.1 nL/ min for 15 s, 30 s and 60 s. The products of this reaction were analyzed by Q1 scan (ESI, positive mode) on a micrOTOF-Q II (Bruker Daltonik, GmbH, Bremen, Germany) in the presence of Na^+^ alkali metal (introduced via a post column consisting of 0.2 mM sodium acetate in methanol at 0.1 mL/min), and LC–MS on the same apparatus (mobile phase: methanol/water 90:10; column: COSMOSIL 5C18-MS-II (2.1 × 250 mm, NACALAI TESQUE, INC., Kyoto, Japan); flow rate: 0.2 mL/min; oven temperature: 40 °C; wavelength: 210 nm). Detailed results are presented in the ESI.

The aim of the of the aforementioned series of experiments was to identify the precise factors associated with the mechanism of SQ-OOHs’ oxidation to SQ hydroperoxy cyclic peroxides (in particular, of 6-OOH-SQ to 2-OOH-3-(1,2-dioxane)-SQ in total SQ-OOHs). However, these findings alone are not sufficient, further evidence is required to elucidate the exact underlying oxidation mechanism. Therefore, an investigation of the intermediate radical species formed throughout this reaction was carried out next.

### Characterization of the intermediate radical species generated during the formation of SQ hydroperoxy cyclic peroxides: case of 2-OOH-3-(1,2-dioxane)-SQ from SQ-OOHs

#### ESR Instrumentation and measurement conditions

A JES-X330 ESR model from JEOL RESONANCE Inc. (Tokyo, Japan) was used with a flat type cell (JEOL ES-LC12) for samples’ introduction in all experiments. The ESR parameters were as follows: sweep time: 30 s; center field: 336 mT; sweep width: 7.5 mT; modulation frequency: 100 kHz; modulation width: 1; amplitude: 100; time constant: 0.03 s; microwave frequency: 9426.24 MHz; microwave power: 2 mW; microwave phase: 750; microwave coupling: 346. A JEOL ES-UXL (with a xenon lamp) irradiator of 60 Klux intensity was used for light-exposed experiments. A UV filter with a wavelength of 360 nm was used for UV irradiation experiments. Mn^2+^ was used as an internal standard in all measurements.

#### Samples’ preparation and analysis of the radicals formed during SQ-OOHs oxidation

**Figure 2:**
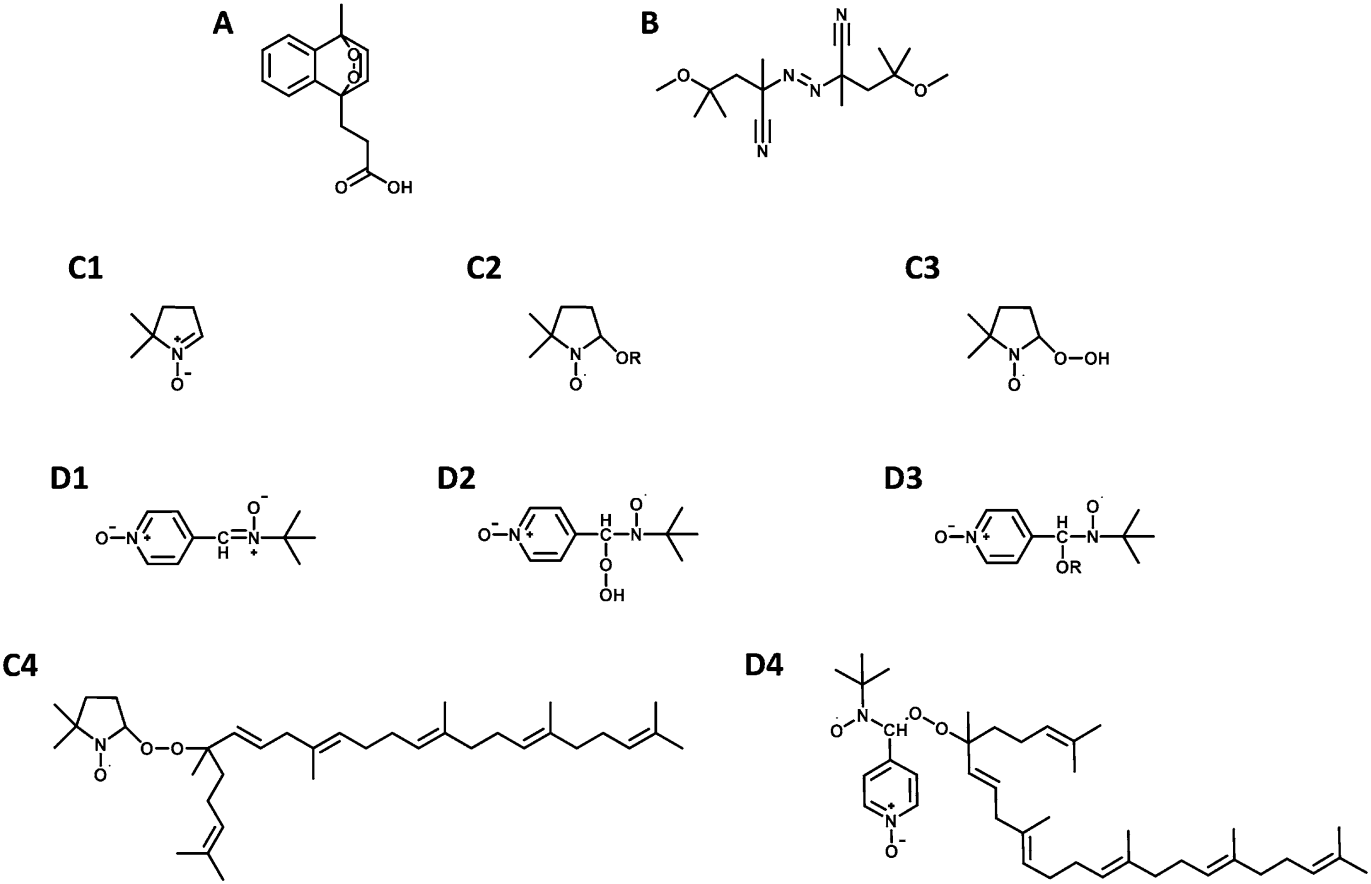
Chemical structures of (**A**) EP, (**B**) MeO-AMVN, (**C1-4**) DMPO and its respective radical adducts, (**D1-4**) POBN and its respective radical adducts.

The spin traps DMPO (Fig. 2C1), and POBN (Fig. 2D1) were used to detect and identify the radicals formed during the oxidative transformation of 6-OOH-SQ to 2-OOH-3-(1,2-dioxane)-SQ in total SQ-OOHs mixture. Interpretation of the hyperfine splitting, comparison of the radicals’ spectra obtained during the formation of 2-OOH-3-(1,2-dioxane)-SQ to the spectra obtained from the radical references (HOO^•^, HO^•^, and O_2_^•−^), and to the literature were carried out to identify the radical species. The experimental design was as follows: DMPO and POBN were each dissolved at 66.7 mg/mL in 0.1 M borate buffer (pH = 9.8). Radical reference of O_2_^•−^ was prepared as reported in a previous study by Samuel E.L.G. et al.^23^. In brief, in an amber glass vial, 600 mg of 18-Crown-6 was dissolved in 10 mL of DMSO to which 71.1 mg of KO_2_ was added. The mixture was stirred for 30 min until a clear pale yellow solution was obtained. The stock solution was used directly or after storage at − 80 °C for a maximum of 14 days. Whereas HOO^•^ and HO^•^ radicals were generated by UV irradiation of H_2_O_2_ and tBuOOH respectively. To 150 mM of either H_2_O_2_ or tBuOOH in borate, was added 40 µL of either DMPO or POBN, vortexed, then exposed to a 360 nm UV light. The reaction was monitored for 30 min and spectra were registered every 30 s. Under alkaline conditions, H_2_O_2_ has been reported by several studies to undergo deprotonation followed by the formation of mainly peroxyl radicals, and minorly hydroxyl radicals^24–26^. The latter would further react with the intact H_2_O_2_ to give HOO^•^ and H_2_O^27^, making HOO^•^ the dominating radical from the UV decomposition of H_2_O_2_ in the present conditions. Whereas tBuOOH was experimentally found to generate HO^•^ and ^•^OR radicals under the described protocol. In the case of SQ-OOHs, a stock solution of the mixture of pure SQ-OOHs was used at a concentration of 150 mM stored in methanol in amber vials purged with N_2_ at − 80 °C. Prior to ESR analyses, the methanol dissolved pure isomers were evaporated then dissolved in 40 µL hexane, to which 120 µL of isopropanol and 40 µL of either DMPO or POBN were added. The mixture was vortexed and introduced directly into the ESR flat tube then exposed to an LED light of 60 Klux intensity overnight (13 h and 30 min). The radicals’ formed were monitored every 30 s. Furthermore, to verify the involvement of O_2_^•−^ in the mechanism, the same oxidation reaction of SQ-OOH/DMPO was performed in the presence of SOD at a final quantity of 44 µg and analyzed by ESR under the same conditions for the same period of time.

### Chemical calculation of the electrostatic charges of SQ-OOHs’ hydroperoxide moiety

Based on our previous study^21^, we found that 6-OOH-SQ and 10-OOH-SQ, in particular, were the first targets of this unknown type of oxidation, followed by 2-OOH-SQ. This targeting of tertiary hydroperoxides in particular upon exposure to photons is believed to be of interest in understanding the oxidative mechanism leading to SQ hydroperoxy cyclic peroxides. One reason for this targeting might be the difference in the distribution of electrostatic charges of the hydroperoxide moiety among the isomers. In order to clarify this observation, calculations of the hydroperoxides’ positive (-H) and negative (–O–O–) electrostatic charges were carried out on Spartan’18 (Wavefunction Inc., Irvine, CA, USA) software (equilibrium geometry/PM3). The negative charges were calculated by summing the two oxygens’ atomic electrostatic charges, and the differences between the negative and positive moieties of each SQ-OOH isomer were compared.

### Data processing

LC–MS/MS data were processed using the Analyst software with manual corrections of the peak area. Plotting of the external standard curve and calculation of the concentrations of 2-OOH-3-(1,2-dioxane)-SQ were carried out on Microsoft Excel. Data were expressed as the mean ± SD, n = 3. ESR and FP8700 data were processed using OriginPro software.

## Results and discussion

### Determination of the optimal oxidative conditions involved in the formation of 2-OOH-3-(1,2-dioxane)-SQ: exposure of SQ-OOHs to different oxidative conditions

**Figure 3:**
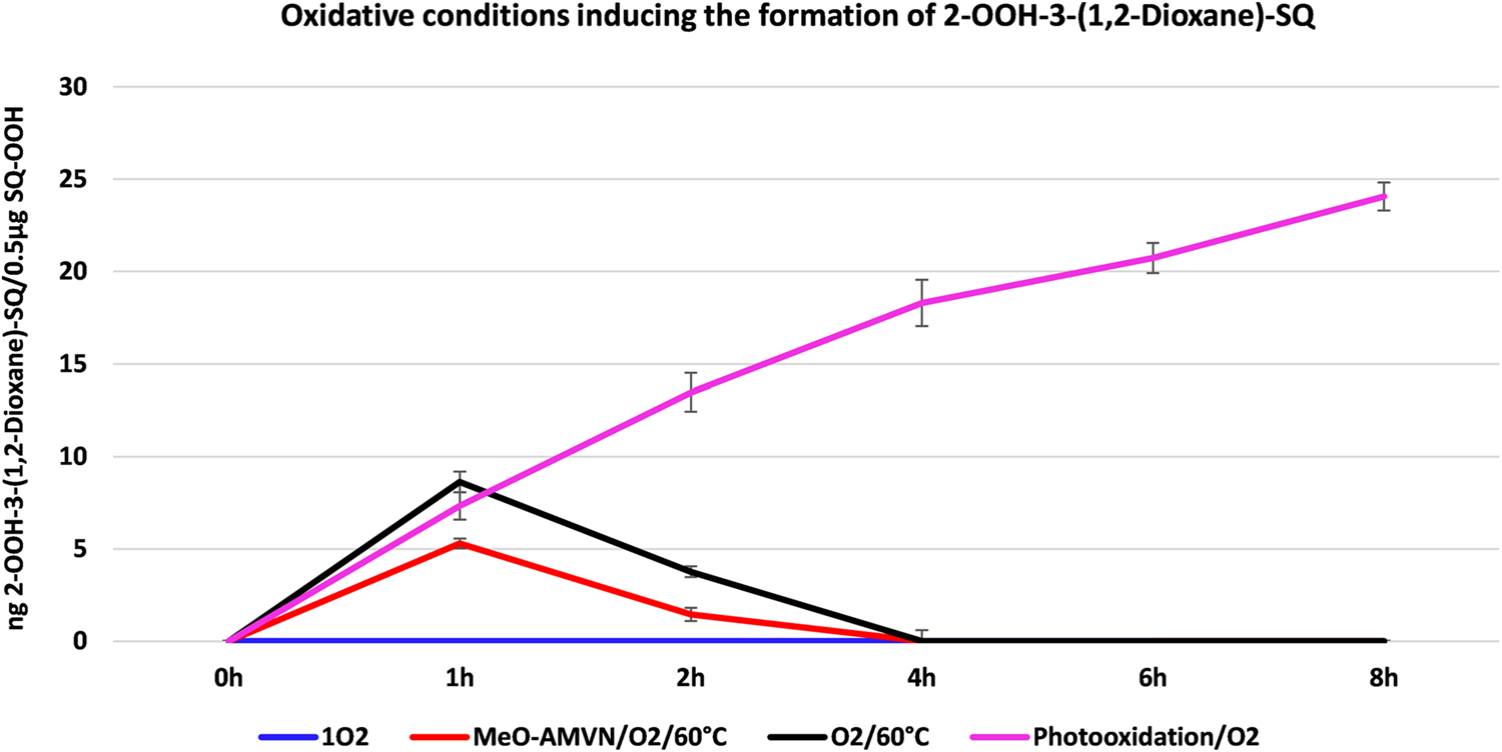
2-OOH-3-(1,2-dioxane)-SQ generation under the different oxidative conditions: ^1^O_2_, MeO-AMVN/O_2_/60°C, O_2_/60°C and Photooxidation/O_2_. Data expressed as mean ± SD, n = 3.

EP (Fig. 2A) is a well-known ^1^O_2_ source with quick and high yields^28,29^. In the experiment where SQ-OOHs were exposed to ^1^O_2_ resulting from the decomposition of EP, the formation of 2-OOH-3-(1,2-dioxane)-SQ was not observed over 8 h (Fig. 3). The absence of 2-OOH-3-(1,2-dioxane)-SQ indicates that ^1^O_2_ is neither a contributing factor nor an initiator in the mechanism involved in the formation of SQ hydroperoxy cyclic peroxides from SQ-OOHs. LC-UV chromatograms of SQ-OOHs showed that the intensity of their peaks barely decreased over a prolonged period of 72 h of incubation with EP at 25 °C with no sign of 2-OOH-3-(1,2-dioxane)-SQ’s peak, which is usually registered around 21.9 min^21^ (Fig. S2, ESI), further confirming their non-reactivity with ^1^O_2_. The generation of ^1^O_2_ was investigated from the decomposition of EP in the presence and absence of SQ-OOHs in deuterated chloroform/methanol (1:1) over 1 h with spectra taken at 5 min intervals. In the absence of SQ-OOHs the formed ^1^O_2_ was constant from 0 to 1 h (Fig. S3A, ESI), in their presence however, ^1^O_2_ was not detected over the same period of time (Fig. S3B, ESI). To better elucidate the absence of ^1^O_2_ signal in the presence of SQ-OOHs, the changes occurring on EP were investigated using LC-UV, Q1 MS scan, and ^1^H NMR. Changes in EP’s LC-UV peaks in the presence and absence of SQ-OOHs gave similar patterns (Fig. S4, ESI), indicating the decomposition of EP under both conditions. Q1 scans of EP’s peaks (Figs. S5 to S8, ESI) show at 0 h the presence of both intact and decomposed EP, while they show mostly fully decomposed EP in the presence and absence of SQ-OOHs after incubation at 25 °C from 1 to 18 h. Likewise, ^1^H NMR spectra interpretation of EP (Table S1 and Figs. S9 to S11, ESI) in the presence and absence of SQ-OOHs show evidence of EP’s decomposition in both conditions, further confirming the generation of ^1^O_2_ in both cases. The absence of ^1^O_2_ phosphorescence peaks in the presence of SQ-OOHs is not well understood and may be either somehow quenched, or due to the presence of trace amounts of radical decomposition species generated from SQ-OOHs at 25 °C. The interaction of these radicals with EP would induce in parallel, a radical decomposition mechanism of EP, generating ^3^O_2_ instead of ^1^O_2_ (Fig. S12, ESI), making the quantity of ^1^O_2_ less and more difficult to detect. Nevertheless, we consider these results to be sufficient evidence to refute the involvement of ^1^O_2_ in the generation of SQ hydroperoxy cyclic peroxides from SQ-OOHs.

Under elevated temperatures, both in the presence and absence of the radical initiator MeO-AMVN (Fig. 2B), the generation of 2-OOH-3-(1,2-dioxane)-SQ was observed, exhibiting an initial increase reaching 5.30 ± 0.27 ng and 8.62 ± 0.56 ng respectively after 1 h. Subsequently, the levels gradually decreased and eventually disappeared after 4 h. The overall generated amount was lower in the case of the presence of the radical initiator (MeO-AMVN) (Fig. 3). These observations indicate that the thermal oxidation of SQ-OOHs is not the ideal condition for the generation of SQ hydroperoxy cyclic peroxides due to their instability and fast degradation under heat, nor is it solely instigated or facilitated by a radical attack since MeO-AMVN exhibited a decrease in the formed amount of 2-OOH-3-(1,2-dioxane)-SQ compared to its absence. The reason for the appearance of the compound under thermal oxidation is proposed to be that the reaction requires a certain amount of activation energy; however, under the same conditions, modifications of LOOHs in general can take place and may be more prone to destructive modifications. Breakdown products are more abundant as a result of the homolytic cleavage of hydroperoxides’ O–O bond which is more favored under elevated temperatures specifically^30–32^, explaining the low generated quantity, the fast decrease and disappearance of 2-OOH-3-(1,2-dioxane)-SQ, as its and SQ-OOHs’ O–O bonds undergo cleavage, yielding mostly decomposition products. For instance, the cleavage of peroxide’s O–O bond to give alkoxyl and hydroxyl radicals is often associated with and measured under thermal oxidation^33^. Under photooxidation and in the presence of ^3^O_2_, the generation of 2-OOH-3-(1,2-dioxane)-SQ was noticed to be the highest among all conditions and continued to increase up until 8 h reaching an amount of 24.07 ± 0.76 ng (Fig. 3). Further confirming that the oxidation mechanism leading to the formation of SQ hydroperoxy cyclic peroxides from SQ-OOHs is photochemically favored. Overall, we noticed that higher molecular weight secondary oxidation products are more likely to be formed under photooxidation with minor breakdown products, while the latter are the major observed secondary oxidation products under thermal oxidation. This further indicates that under the present conditions, thermal oxidation of SQ-OOHs has a higher tendency for “destructive modifications” while their photooxidation has more of “constructive modifications” pattern.

Both unoxidized SQ and SQ-OOHs’ exposure to ozone resulted mainly in breakdown products, 2-OOH-3-(1,2-dioxane)-SQ was not one of the major products in both cases (Fig. S13, ESI). Hence, ozone, which is in constant contact with human skin lipids, especially in polluted environments, has no role or effect on the mechanism generating 2-OOH-3-(1,2-dioxane)-SQ from SQ-OOHs or directly from SQ.

From the above set of comparative oxidative conditions, we could confirm that the serial cyclization of SQ-OOHs to give SQ hydroperoxy cyclic peroxides is ideally induced and generated by photooxidation in the presence of ^3^O_2_, making them the optimal conditions for this reaction.

### Characterization of the intermediate radical species generated during the formation of SQ hydroperoxy cyclic peroxides

**Figure 4:**
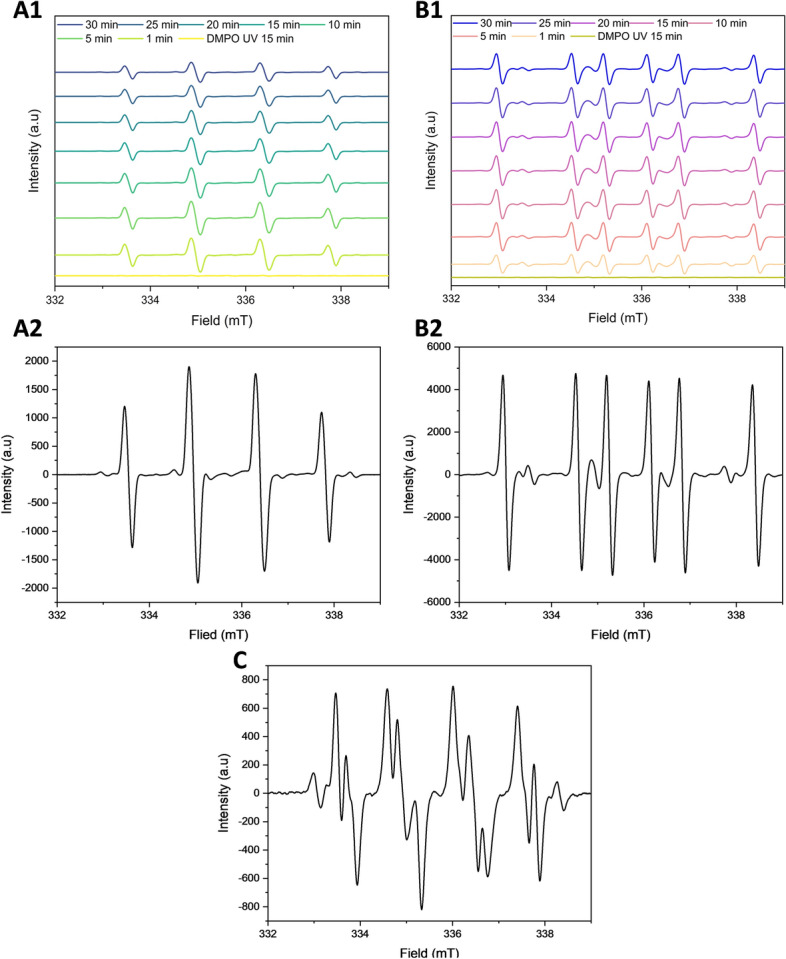
ESR spectra of (**A1**) tBuOOH over 30 min under UV, (**A2**) individual tBuOOH under UV, (**B1**) H_2_O_2_ over 30 min under UV, (**B2**) individual H_2_O_2_ under UV, and (**C**) O_2_^•−^ generated from KO_2_. Using DMPO as the spin trap.

ESR spectroscopy was employed to identify and monitor the formation of radicals during the photooxidation of SQ-OOHs and the generation of SQ hydroperoxy cyclic peroxides, specifically, 2-OOH-3-(1,2-dioxane)-SQ. To identify the radical species formed during the reaction, reference radicals were used, and their spectra, including hyperfine splitting and coupling constants, along with the literature, were compared to those obtained from SQ-OOHs’ oxidation to determine the generated intermediate radical species. Spectra obtained from the UV irradiation of tBuOOH generated ^•^OR and HO^•^ radicals over 30 min (Fig. 4A1,A2) based on the hyperfine splitting interpretation (Fig. S14, ESI), which were determined as DMPO-OH/OR adducts (Fig. 2C2). Its characteristic four peaks of approximately 1:2:2:1 intensities and the coupling constants a^N^ = 14.29 G and a_ß_^H^≈14.21 G were also consistent with the DMPO-OH spectra reported in the literature^34–37^. Spectra obtained from the UV irradiation of H_2_O_2_ in the presence of DMPO over 30 min (Fig. 4B1,B2) generated majorly HOO^•^ (adduct presented in Fig. 2C3) following the interpretation of the hyperfine splitting (Fig. S15, ESI), which was consistent with DMPO peroxyl radical adducts reported in the literature^38–40^, with the coupling constants a^N^ = 15.75 G and a_ß_^H^ = 22.41 G. And minorly HO^•^ with the constants a^N^ = 14.23 G and a_ß_^H^≈14.20 G.

O_2_^•−^ spectrum presented in Fig. 4C (interpretation in Fig. S16, ESI), shows a DMPO-OO^−^ adduct with a similar 12 splitting pattern as reported previously^41,42^.

**Figure 5:**
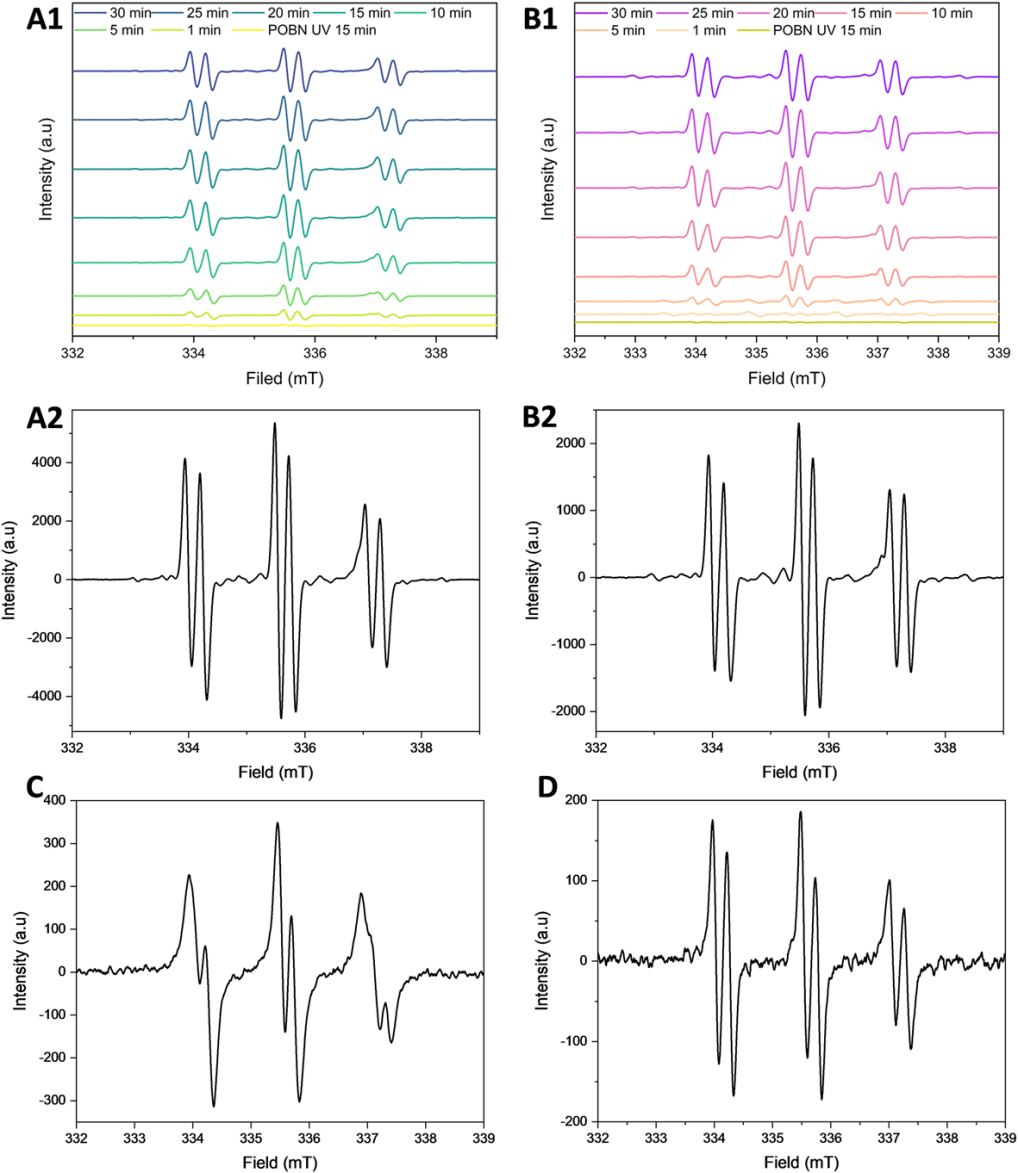
ESR spectra of (**A1**) tBuOOH over 30 min under UV, (**A2**) individual tBuOOH under UV, (**B1**) H_2_O_2_ over 30 min under UV, (**B2**) individual H_2_O_2_ under UV, (**C**) O_2_^•−^ generated from KO_2_, and (**D**) SQ-OOHs photooxidation. Using POBN as the spin trap.

DMPO presented clear characteristic and distinguishable adduct-based hyperfine splitting patterns, depending on the type of the radical. However, this was hardly the case when POBN was used as the spin trap (adducts presented in Fig. 2D2–D4). The spectra shown in Fig. 5 did not exhibit significant differences among the POBN radical adducts that could help identify the radical species. Moreover, over a period of 13 h and 30 min, SQ-OOHs derived radicals quenched by POBN were detected between 0 and 4 h only (Fig. S17, ESI), while they were detected between 0 and 6 h in the case of DMPO with higher intensities. Consequently, DMPO was chosen as the primary spin trap for the identification and analysis of the radicals formed during the cyclization of SQ-OOHs to SQ hydroperoxy cyclic peroxides.

**Figure 6:**
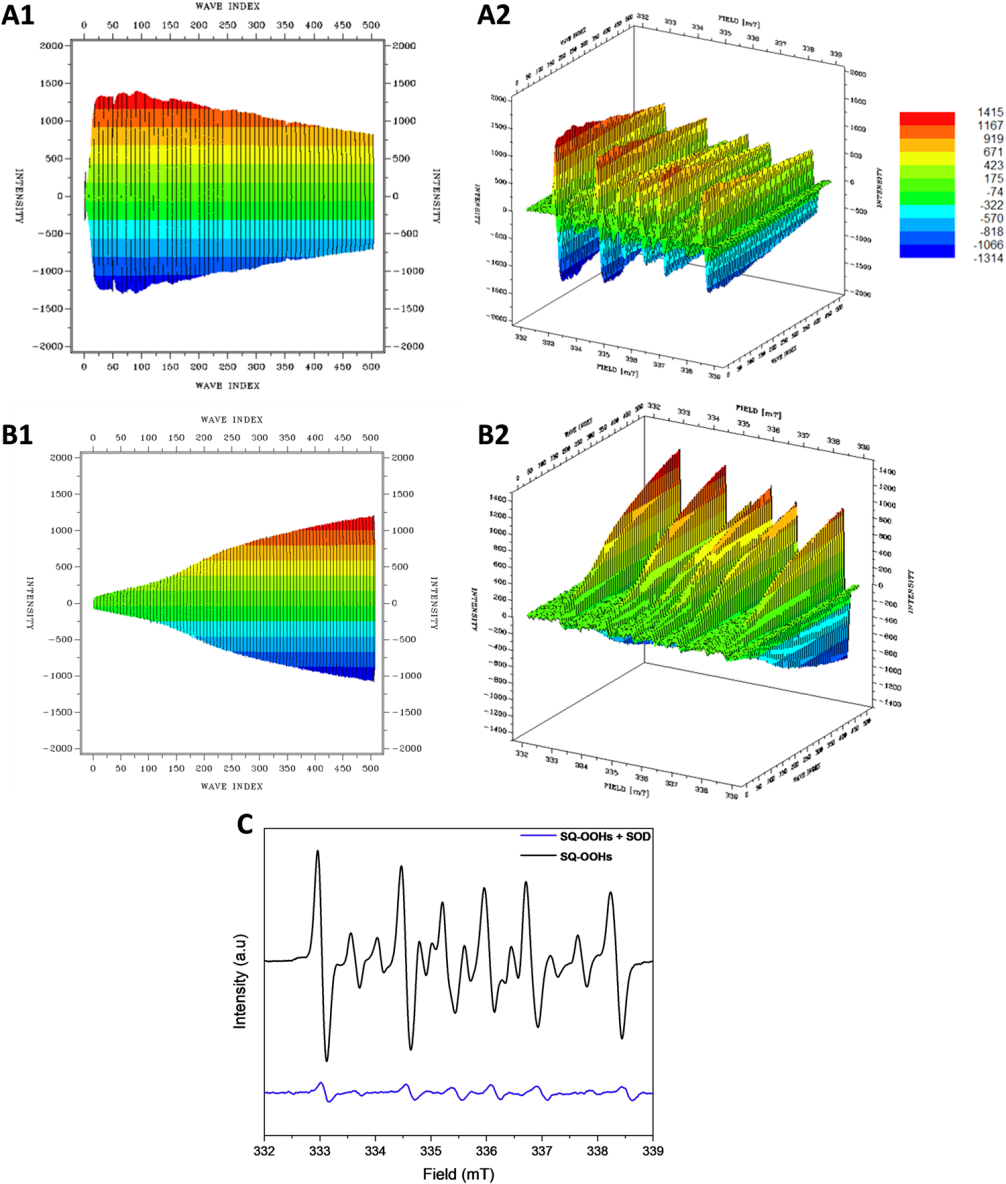
(**A1**) side profile and (**A2**) 3D representations of the DMPO/SQ-OOHs photooxidation spectra generated over 13 h and 30 min. (**B1**) side profile and (**B2**) 3D representations of the DMPO/SQ-OOHs photooxidation spectra generated over 13 h and 30 min in the presence of SOD. (**C**) Comparison of individual spectra of DMPO/SQ-OOHs photooxidation in the presence and absence of SOD after 4h.

**Figure 7:**
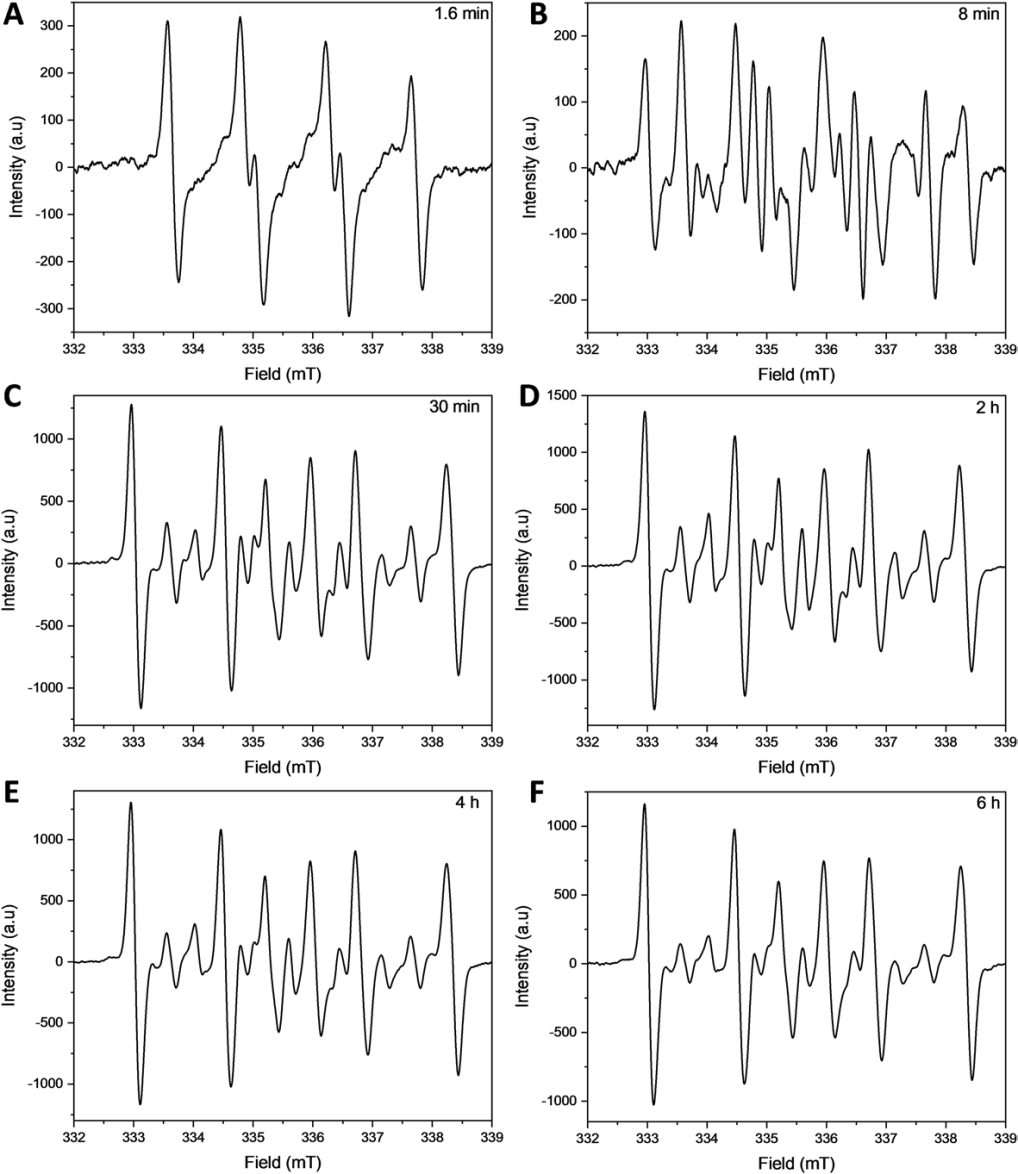
Individual ESR spectra of DMPO/SQ-OOHs photooxidation after (**A**) 1.6 min, (**B**) 8 min, (**C**) 30 min, (**D**) 2 h, (**E**) 4 h, and (**F**) 6 h.

**Figure 8:**
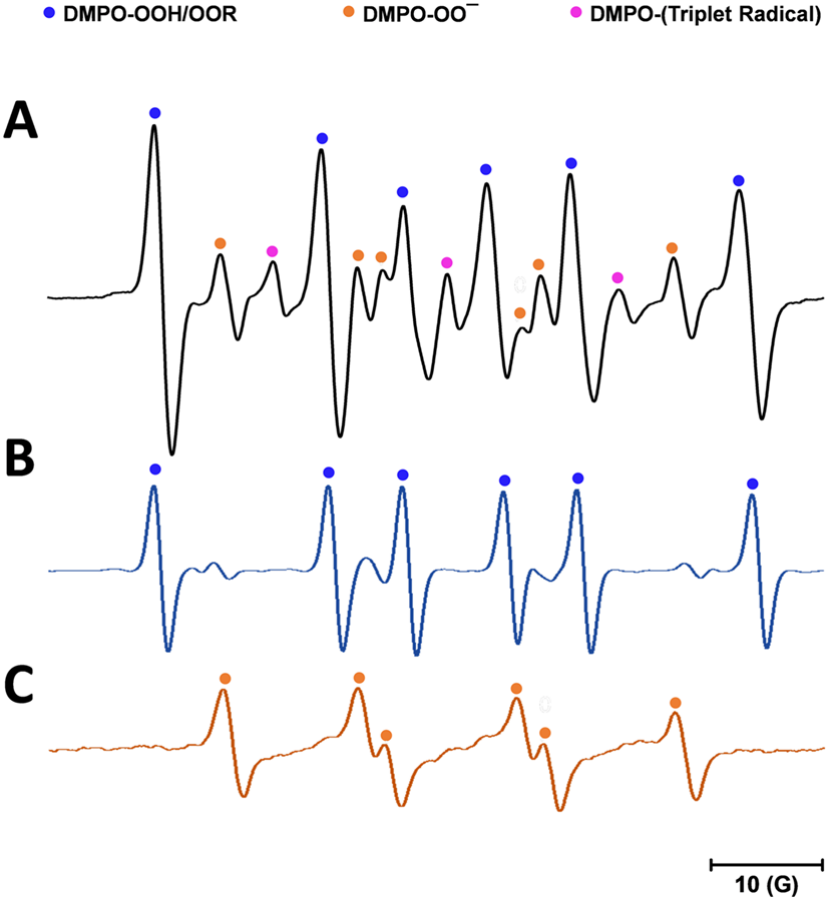
Demonstration of the radicals identified during DMPO/SQ-OOHs photooxidation by comparison of their spectra to DMPO-OOH and DMPO-OO^−^ spectra.

The oxidation of SQ-OOHs in the presence of DMPO provided significant information as shown in the side profile and 3D representations of the radicals’ turnover overnight (13 h and 30 min) in Fig. 6A1,A2. Radicals were observed throughout the oxidation process, with the highest intensities between 0 and 6 h. A distinct change in the pattern of the radicals was observed exclusively during the initial 12.8 min, to give similar constant spectra for the rest of the oxidation (Fig. 6). At 1.6 min, the registered spectrum (Fig. 6A) was compatible with DMPO-OO^−^ reported in several previous studies^43–45^. Its hyperfine pattern appeared to differ from the one obtained from the pure O_2_^•−^ in Fig. 4, this discrepancy can be attributed to the solvent composition in the case of SQ-OOHs, which contained mainly hexane and isopropyl alcohol instead of DMSO. This is consistent with the previous reports that gave similar spectra when the solvent used contained alcohols^43,44^. Following the initial 1.6 min of photooxidation of SQ-OOHs, the signal of O_2_^•−^ radicals decreased but remained detectable, this can be partially due to its half-life (order of 10^–6^ s) compared to that of ROO^•^ (order of 17 s)^46^. Notably, a prominent hyperfine structure emerged and continued to intensify over time, as observed in Fig. 7B–E. Additionally, another minor unidentified three peaks hyperfine structure of 1:1:1 intensities and the coupling constant: a^N^ = 15.48 G (Fig. 8) was registered. A similar observation was reported by Walger et al. in their investigation of the radicals generated from H_2_O_2_/Cu^II^/phenanthroline system^47^, where the hyperfine structure could also not be identified and was referred to as “triplet radical”. The major hyperfine structure was consistent with DMPO-OOH and DMPO-OOR (i.e. SQ-OO^•^ radicals, adduct with DMPO presented in Fig. 2C4) with the coupling constants a^N^ = 15.20 G and a_ß_^H^ = 22.65 G (Fig. 8), which are very close to the coupling constants of DMPO-OOH observed from the photolysis of H_2_O_2_ (a^N^ = 15.75 G and a_ß_^H^ = 22.41 G) indicating the presence of both SQ-OO^•^ and HOO^•^ radicals. The slight differences can be explained by both the effect of the radical’s substitution and the presence of multiple overlapping radical signals in the case of SQ-OOHs photooxidation. Moreover, carbon radicals present similar hyperfine splitting structure with coupling constants that vary depending on the substitution of the radical^48,49^, for this reason, the possibility of the presence of a carbon centered radical from SQ-OOHs during this photooxidation cannot be overruled.

Upon addition of SOD, the duration in which the radicals were observed to form in high intensities in its absence, appeared to have no signal at first or a very low signal (i.e., no radicals) (Fig. 6C) up until 4 h. This is apparent from the comparison of the 3D and side profile data (Fig. 6B1 and B2). SOD is a well-known O_2_^•−^ scavenger, the elimination of radicals’ signals during the peak period of radical formation resulting from the oxidation of SQ-OOHs upon its addition, highly confirms the involvement of O_2_^•−^ as a key first radical intermediate in the serial cyclization of SQ-OOHs to SQ hydroperoxy cyclic peroxides. The pattern of the radicals formed during SQ-OOHs photooxidation in the presence of SOD, which were generated gradually and that could be clearly seen after 4 h, presented one species compatible with the HOO^•^ spectra (Fig. S18, ESI) (which can also be interpreted as ROO^•^ and/or R^•^). Moreover, minor radicals (O_2_^•−^, and triplet radical) previously detected during the oxidation of SQ-OOHs were not observed in the presence of SOD throughout the oxidation. Hence, it can be inferred that the detected radical is solely HOO^•^, which is generated through the photolysis of H_2_O_2_, product of SOD’s O_2_^•−^ quenching activity. Although O_2_^•−^ has been previously hypothesized to be involved in lipid peroxidation by acting both in the initiation and termination steps in in vivo and in vitro studies^50–53^, to the best of our knowledge, there are no reports that demonstrated its implication in the formation of higher molecular weight secondary oxidation products from LOOHs, especially, in their serial cyclization. We, therefore, present the first evidence of the crucial involvement of O_2_^•−^ in the formation of SQ hydroperoxy cyclic peroxides from SQ-OOHs. Furthermore, LC–MS/MS analysis of 2-OOH-3-(1,2-dioxane)-SQ generated from the photooxidation of SQ-OOHs in the presence of SOD (Fig. S19, ESI) showed no formation of 2-OOH-3-(1,2-dioxane)-SQ and a decline in the starting trace amount of the secondary oxidation product. This further confirms that SOD suppresses the generation of 2-OOH-3-(1,2-dioxane)-SQ and that O_2_^•−^ plays a critical role in the formation of SQ hydroperoxy cyclic peroxides from SQ-OOHs.

**Figure 9:**
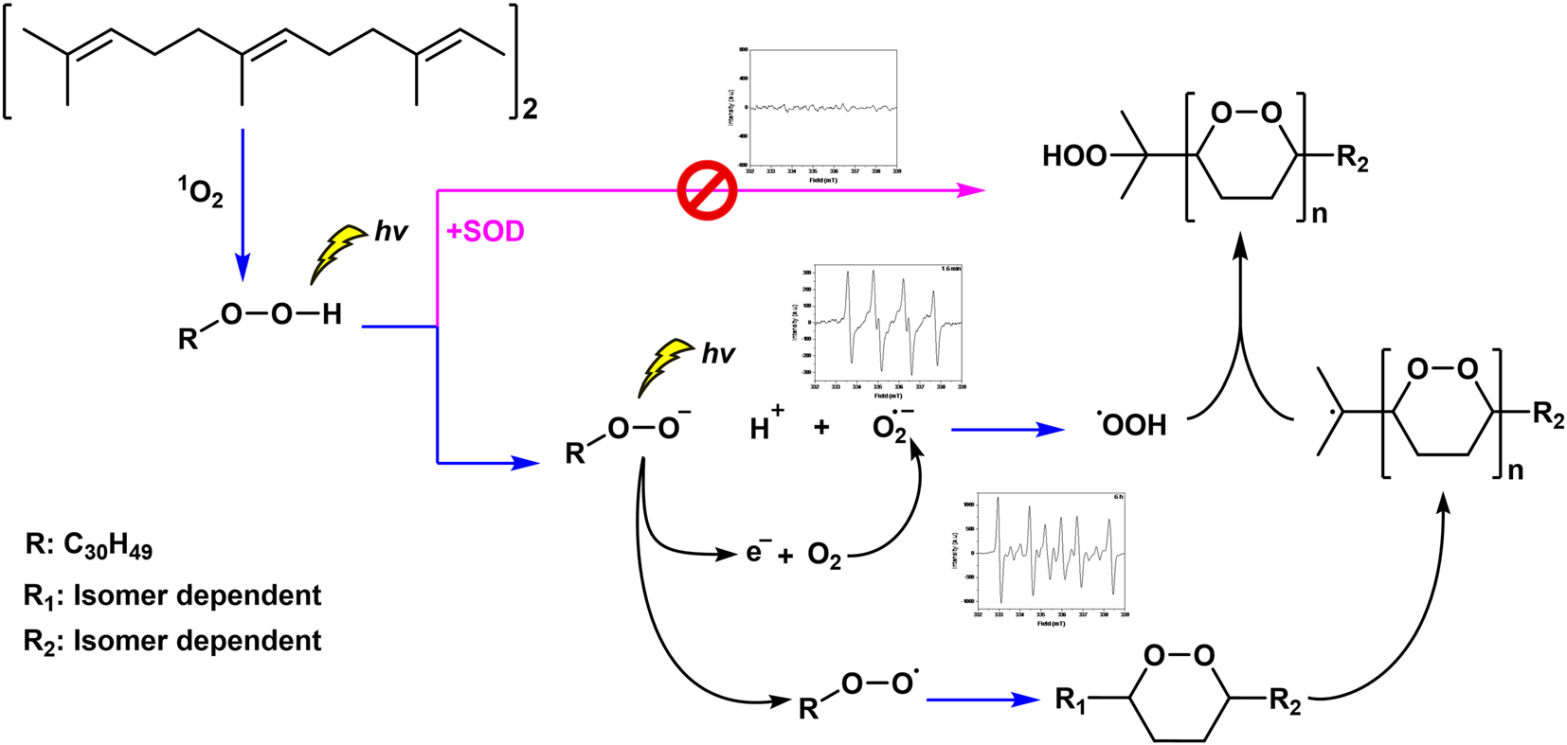
Schematic overview of the proposed mechanism of SQ-OOHs conversion to SQ hydroperoxy cyclic peroxides.

Chemical calculations of the O–O’s overall negative electrostatic charge and the proton’s positive charge of each isomer’s hydroperoxide’s moiety as determined by Spartan 18 software are expressed in Table 1. The difference between the negative and positive electrostatic charges was found to be most pronounced in the isomers 6-OOH-SQ, 10-OOH-SQ and 2-OOH-SQ with the values: − 0.296, − 0.292 and − 0.293 respectively. This indicates that tertiary SQ-OOHs exhibit a greater disparity between negative and positive electrostatic charges compared to the secondary isomers (which had the values of − 0.246, − 0.275 and − 0.286). This can be attributed to factors such as hyperconjugation, the inductive effect, and the stabilizing steric effect (reduced steric hindrance) which are more pronounced in the tertiary isomers. The enhanced inductive effect promotes the stabilization of the hydroperoxide-bearing quaternary carbon, as a result, the hydroperoxide moiety of tertiary SQ-OOHs exhibits increased reactivity. Heterolytic cleavage is characterized by a more common occurrence when there is a significant disparity between the negative and positive electrostatic charges at the two ends of a bond. The greater difference in electrostatic charges between the oxygen and hydrogen moieties in tertiary SQ-OOHs allows for a higher likelihood of heterolytic cleavage of the O–H bond, compared to the secondary isomers (Illustration in Fig. S20, ESI), explaining why 6-OOH-SQ and 10-OOH-SQ are the primary targets of this photooxidation mechanism^21^. The above mentioned criteria are all influential factors that contribute to the enhanced stability of tertiary peroxyl radicals. Consequently, tertiary hydroperoxides are more prone to decomposition via heterolytic cleavage than secondary hydroperoxides in a mixture, followed by the release of a photoelectron to produce a more stable peroxyl radical. These factors also affect the geometry of the different isomers^54^, which may, in turn, affect the stability of the resulting ions and radicals, the reaction, and its rate. Notably, this reaction was observed to exhibit significant differences when conducted in different solvents. Specifically, when methanol was used as the solvent for the oxidation of total SQ-OOHs, of which 6-OOH-SQ and 10-OOH-SQ are the first targets, LC-UV analysis revealed no decrease in the peaks’ intensities of these two isomers, the peak corresponding to 2-OOH-3-(1,2-dioxane)-SQ was not detected. In contrast, when the oxidation was carried out in hexane, a noticeable decrease in the peaks’ intensities was observed for the two isomers, accompanied by the appearance of the peak corresponding to 2-OOH-3-(1,2-dioxane)-SQ (Fig. S21, ESI), the observed difference can be attributed to the fact that methanol, unlike hexane, functions as a proton donor, hindering cyclization by facilitating proton addition to the formed SQ-OO^•^ radical. This further highlights the significance of the choice of the reactive conditions on its outcomes. The above O–H heterolytic cleavage route also supports the formation and detection of O_2_^•−^ radicals by ESR as the first radical species formed from the photooxidation of SQ-OOHs. Moreover, Q1 analyses of DMPO adducts with radicals resulting from SQ-OOHs photooxidation and thermal oxidation (Fig. S22, ESI), confirm the presence of both DMPO-O-SQ/OH and DMPO-OO-SQ under thermal oxidation with high decomposition products, while it shows the detection of only DMPO-OO-SQ under photooxidation. Further confirming our proposed O–H cleavage under photooxidation and the statement made in the discussion of SQ-OOHs thermal oxidation in the previous section. Consequently, we suggest the hereinafter mechanism (Fig. 9). In the case of 6-OOH-SQ, following cleavage, the formed SQ-OO^−^ further undergoes release of an electron upon exposure to photons under the described conditions, which is subsequently accepted by ^3^O_2_, generating O_2_^•−^ and SQ-OO^•^. The latter undergoes cycloaddition on the adjacent C3 carbon to give a 1,2-dioxane ring and a carbon radical on C2. While O_2_^•−^ reacts with H^+^ to give a peroxyl radical, which subsequently reacts with the C2 carbon radical to give a hydroperoxide, generating 2-OOH-3-(1,2-dioxane)-SQ. In the case of the remaining isomers, the same mechanism applies with serial cyclization from the firstly formed SQ-OO^•^ to give multiple 1,2-dioxane rings and a hydroperoxide on C2 (as shown in Fig. 1). While prior investigations have proposed the initiation of the serial cyclization in the formation of lipid cyclic peroxides from LOOH via the generation of a peroxyl radical (LOO^•^) and a proton radical (H^•^)^8–16^, the actual formation mechanisms of these radicals have not been substantiated or demonstrated. In the current study, we present compelling proof regarding the unforeseen participation of O_2_^•−^ in the formation of these radicals, the serial cyclization of SQ-OOH, and the strongly probable heterolytic cleavage of the O–H bond as the initial step in this reaction under photocatalytic conditions.

**Table 1:** Chemical calculations of the difference between the negative electrostatic charges (–O–O–) and positive electrostatic charges (–H) of SQ-OOHs hydroperoxide moiety.

Electrostatic charges						
Isomer	11-OOH-SQ	10-OOH-SQ	7-OOH-SQ	6-OOH-SQ	3-OOH-SQ	2-OOH-SQ
R–(O–O)–H	− 0.612	− 0.652	− 0.64	− 0.659	− 0.59	− 0.65
R–O–O–(H)	0.337	0.36	0.354	0.363	0.344	0.357
Charge difference	− 0.275	− 0.292	− 0.286	− 0.296	− 0.246	− 0.293

In the present work, it was observed that despite the general misconception arising from the generalization of the values of bond dissociation energies (BDEs), which would indicate a higher likelihood of O–O homolytic cleavage, the cleavage of O–H bonds, both heterolytic and homolytic, is not inherently forbidden by any laws. In fact, based on the totalitarian principle^55^, it is still quite probable, which is what was observed in our case study. Moreover, although heterolytic cleavage energies are in general considered to be higher than BDEs for the same type of bond, the accurate bond cleavage pathway may not always abide by the values provided in the literature, as BDEs and bond ionization energies (BIEs) are estimated calculative and experimental approximations under certain reactive conditions, and may not reflect the behavior of molecules in different experimental procedures. Changing one parameter, such as the solvent, which can introduce a distinct cage effect, has the potential to significantly influence the dynamics of the reactive molecules, resulting in unexpected patterns of bond cleavage, reaction outcomes, and rates^56–59^. This highlights that the general applicability of BDEs and BIEs for a specific bond cannot be assumed ipso facto based on the values available in the literature, unless calculated under identical reactive conditions. This becomes particularly important when investigating unfamiliar reaction mechanisms; hence, these values should be regarded as circumstantial rather than definitive. Several studies have in fact pointed out the limitations and deficiencies associated with this prevailing misapprehension, as they demonstrated different bond cleavage values and reaction pathways based on the specific reaction’s conditions and substitutions of the functional group in question for a variety of compounds^33,60–63^.

## Conclusion

The results presented herein take us a few steps closer to fully understanding and unravelling what is believed to be a novel oxidation mechanism involved in the formation of lipid hydroperoxy cyclic peroxides from the photooxidation of LOOHs. In the present case, we studied how SQ-OOHs give rise to one particular cyclic peroxide (2-OOH-3-(1,2-dioxane)-SQ) from 6-OOH-SQ specifically. We found that the reaction is photochemically favored in the presence of ^3^O_2_, with the following radicals being the prevailing intermediate species: O_2_^•−^, SQ-OO^•^, and HOO^•^. We also demonstrated a link between SQ-OOH tertiary isomers being the first targets of this photooxidation mechanism, and high values of the difference in the electrostatic charges between the positive and negative moieties of their hydroperoxide groups compared to secondary SQ-OOHs. Based on the above evidence, we suggested a reaction mechanism leading to the formation of SQ hydroperoxy cyclic peroxides from SQ-OOHs initiated by photoinduced heterolytic cleavage of the hydroperoxide’s O–H bond in hexane, followed by the formation of O_2_^•−^ and SQ-OO^•^ upon the release of a photoelectron from the anionic form of the latter. HOO^•^ is formed next from the reaction between O_2_^•−^ and H^+^, which subsequently reacts with the carbon radical resulting from the cyclization of SQ-(OO)_n_-OO^•^ on the γ double bond in the last step of this serial cyclization.

These findings pave the way for future investigations aiming to extend the herein proposed serial cyclization mechanism to LOOH compounds in general under similar conditions. Such studies have the potential to enhance our understanding of the impact and implications of lipid hydroperoxy cyclic peroxides in living organisms, as knowledge of the mechanism would enable the facilitation or inhibition of their formation to study their effects and mitigate any negative consequences that they may implicate. Moreover, this mechanism holds promise as a valuable tool in the field of organic synthesis.

### Supplementary Information

Supplementary Information.

## Data Availability

Data is available in the article and supplementary information. (Raw data can be obtained upon reasonable request from the corresponding author).
