# Supplementary material for: Unveiling an unexpected superoxide-mediated photooxidation mechanism of squalene monohydroperoxides to squalene hydroperoxy cyclic peroxides through ESR and LC–MS/MS analyses
Source: Sci Rep. 2023 Nov 9;13:19525. doi: 10.1038/s41598-023-46044-9 (PMC10636020; doi:10.1038/s41598-023-46044-9)
Supplement: Supplementary file 1 — Supplementary Information. [file 41598_2023_46044_MOESM1_ESM.pdf]

Supplementary information

Unveiling an unexpected superoxide-mediated photooxidation mechanism of squalene monohydroperoxides to squalene hydroperoxy cyclic peroxides through ESR and LC-MS/MS analyses

Saoussane Khalifa,[a] Masaru Enomoto,[b] and Kiyotaka Nakagawa[*a]

[a] Laboratory of Food Function Analysis, Graduate School of Agricultural Science, Tohoku University, Sendai 980-8572, Japan

[b] Applied Bioorganic Chemistry Laboratory, Graduate School of Agricultural Science, Tohoku University, Sendai 980-8572, Japan

[*]Corresponding authors:

Prof. Kiyotaka Nakagawa

E-mail: kiyotaka.nakagawa.c1@tohoku.ac.jp

Contents

1- $^{1}O_2$ generation from RB in H_2O, D_2O, and isopropanol analyzed by its phosphorescence............. 1

2- LC-UV analysis of SQ-OOHs in the presence and absence of EP over 72 h. 2

3- $^{1}O_2$ generation from EP in the absence and presence of SQ-OOHs. 3

4- LC-UV analysis of EP in the absence and presence of SQ-OOHs over 18 h. 4

5- Q1 MS analysis of individual EP peaks collected from its LC-UV separation. 5

6- ^{1}H NMR analyses of EP's decomposition in the absence and presence of SQ-OOHs. 9

7- Proposed EP decomposition mechanisms in the absence and presence of SQ-OOHs. 11

8- Q1 MS and LC-MS analyses of SQ and SQ-OOHs products upon exposure to ozone. 12

9- Interpretation of DMPO/tBuOOH ESR spectrum upon UV irradiation. 13

10- Interpretation of DMPO/H_2O_2 ESR spectrum upon UV irradiation. 14

11- Interpretation of DMPO/ $O_2^{\bullet-}$ spectrum obtained from KO_2. 15

12- Side profile representation of POBN/SQ-OOHs photooxidation. 16

13- ESR spectra comparison of DMPO/SQ-OOHs/SOD photooxidation over time with DMPO/SQ-OOHs main spectrum and DMPO/H_2O_2. 17

14- LC-MS/MS analysis of 2-OOH-3-(1,2-dioxane)-SQ generated from the photooxidation of SQ-OOHs in the presence of SOD. 18

15- Schematic interpretation of the inductive effect's role in the targeting of tertiary SQ-OOHs compared to secondary SQ-OOHs. 19

16- LC-UV analysis of the solvent effect on the photooxidation of SQ-OOHs and the generation of 2-OOH-3-(1,2-dioxane)-SQ. 20

17- Q1 MS analysis of the DMPO adducts formed during SQ-OOHs' photooxidation and thermal oxidation. 21

1- $^1O_2$ generation from RB in H_2O, D_2O, and isopropanol analyzed by its phosphorescence.

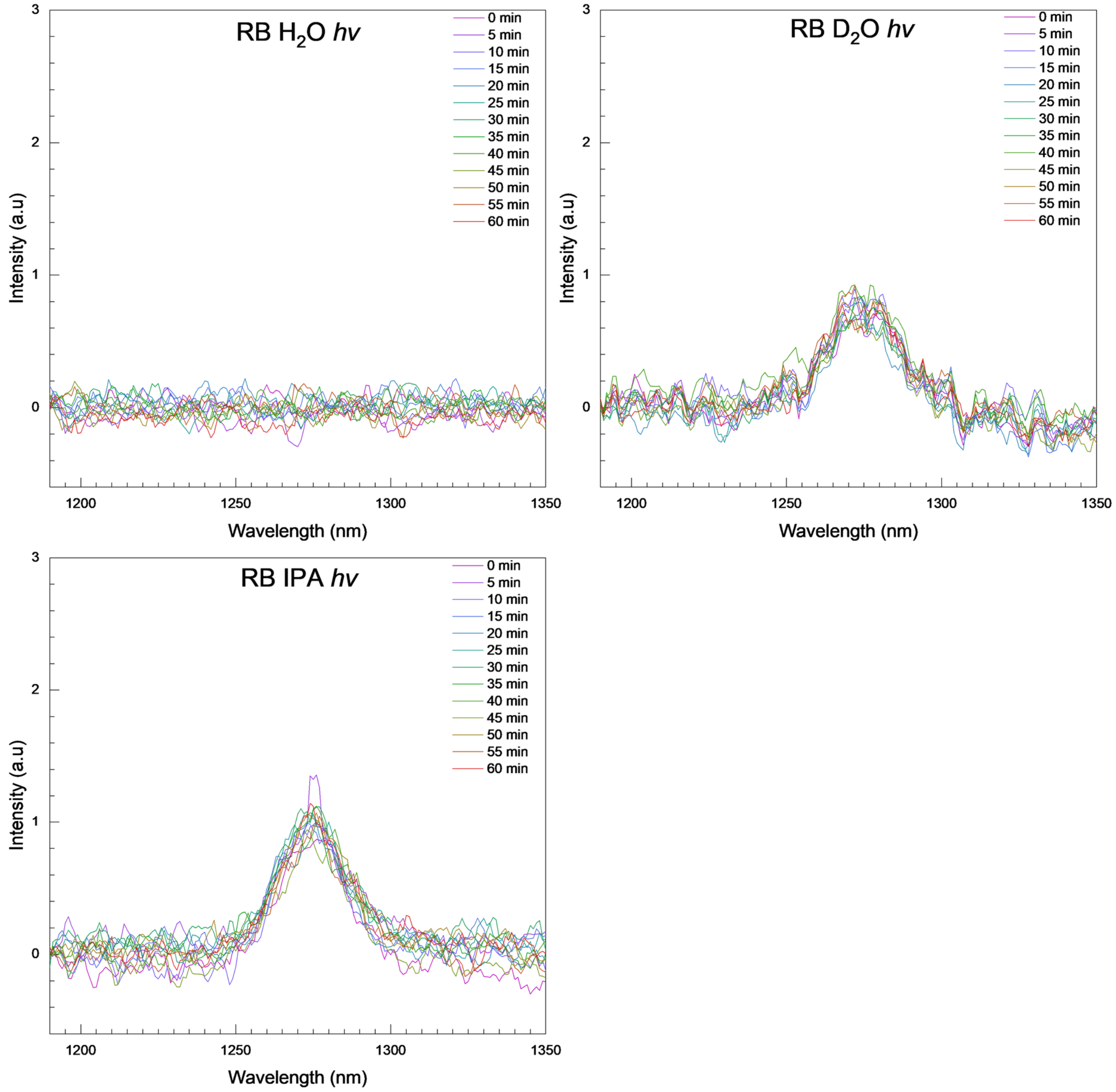

Fig. S1. $^1O_2$ generation over 60 min from RB in H_2O (A), D_2O (B), and isopropanol (IPA) (C), measured by its phosphorescence at 1280 nm (FP8700 apparatus). During the reaction with SQ, RB is dissolved primarily in H_2O. D_2O was used since the lifetime of $^1O_2$ is known to be longer in deuterated solvents, permitting the confirmation of the generation of $^1O_2$ in H_2O. Interestingly, the intensity of $^1O_2$ signal was higher when RB was dissolved in IPA, this information is particularly useful when the reaction's main solvent is not miscible with water, RB can be dissolved in IPA while guaranteeing the generation of an equal, not to say a greater amount of $^1O_2$.

2- LC-UV analysis of SQ-OOHs in the presence and absence of EP over 72 h.

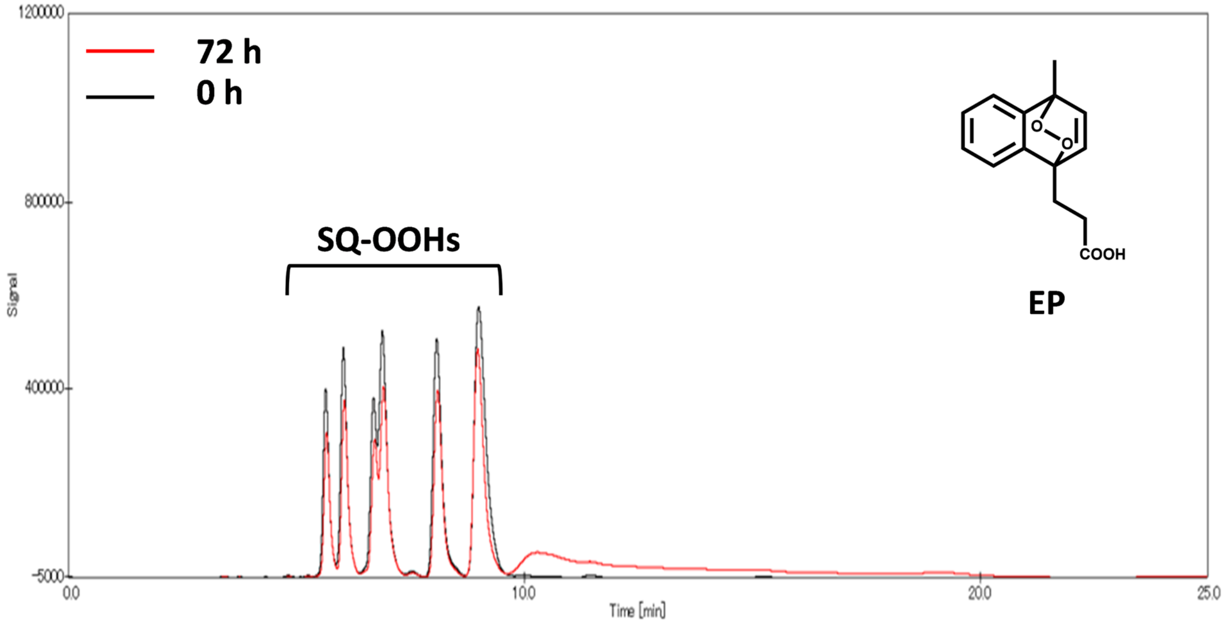

Fig. S2. Changes occurring on SQ-OOHs LC-UV peaks in normal phase in the presence of EP over 72 h. The slight decrease in the intensity of the peaks is believed to be due to the formation of volatile decomposition products arising from SQ-OOHs radical decomposition which is inevitable at room temperature.

3- $^{1}O_2$ generation from EP in the absence and presence of SQ-OOHs.

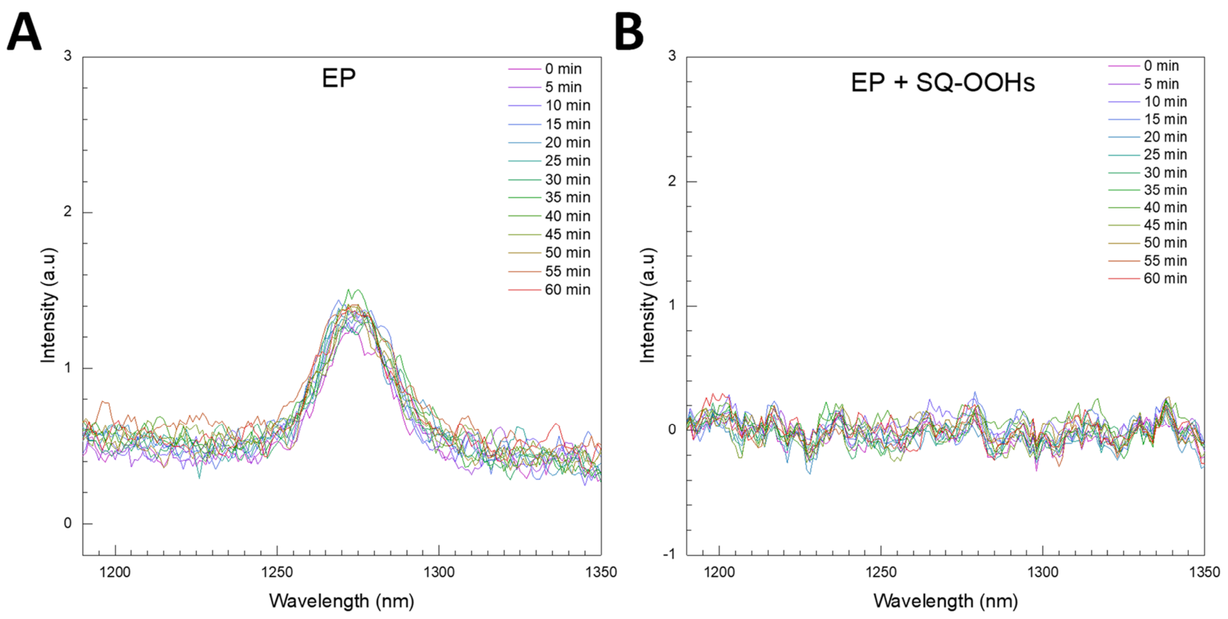

Fig. S3. Generation of $^{1}O_2$ measured by its phosphorescence at 1280 nm (FP8700 apparatus) from the decomposition of 4 mg of EP in the absence (A) and presence of SQ-OOHs (B) compared over 1 h.

4- LC-UV analysis of EP in the absence and presence of SQ-OOHs over 18 h.

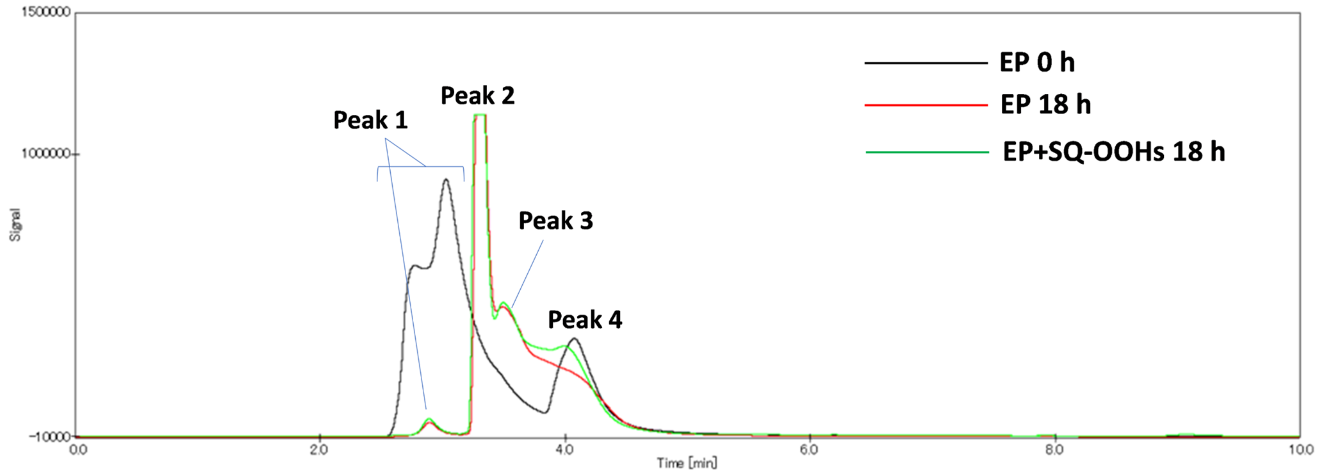

Fig. S4. LC-UV chromatograms of EP at 0 h, EP or EP+SQ-OOHs after a prolonged period of 18 h of incubation at 25°C. Peaks from 1 h to 18 h showed the same pattern.

5- Q1 MS analysis of individual EP peaks collected from its LC-UV separation.

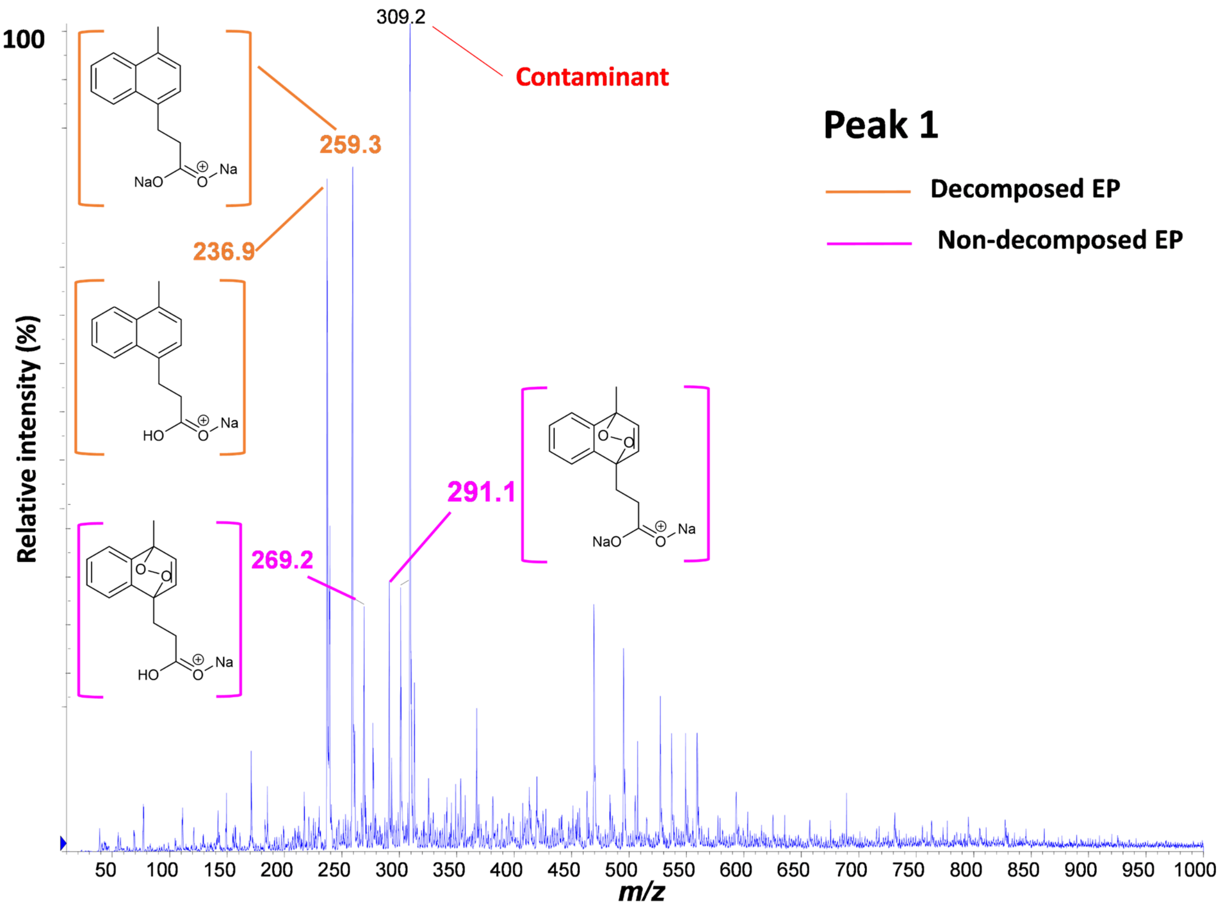

Fig. S5. Q1 scan of the "Peak 1" indicated in the LC-UV chromatograms of EP (Fig. S4).

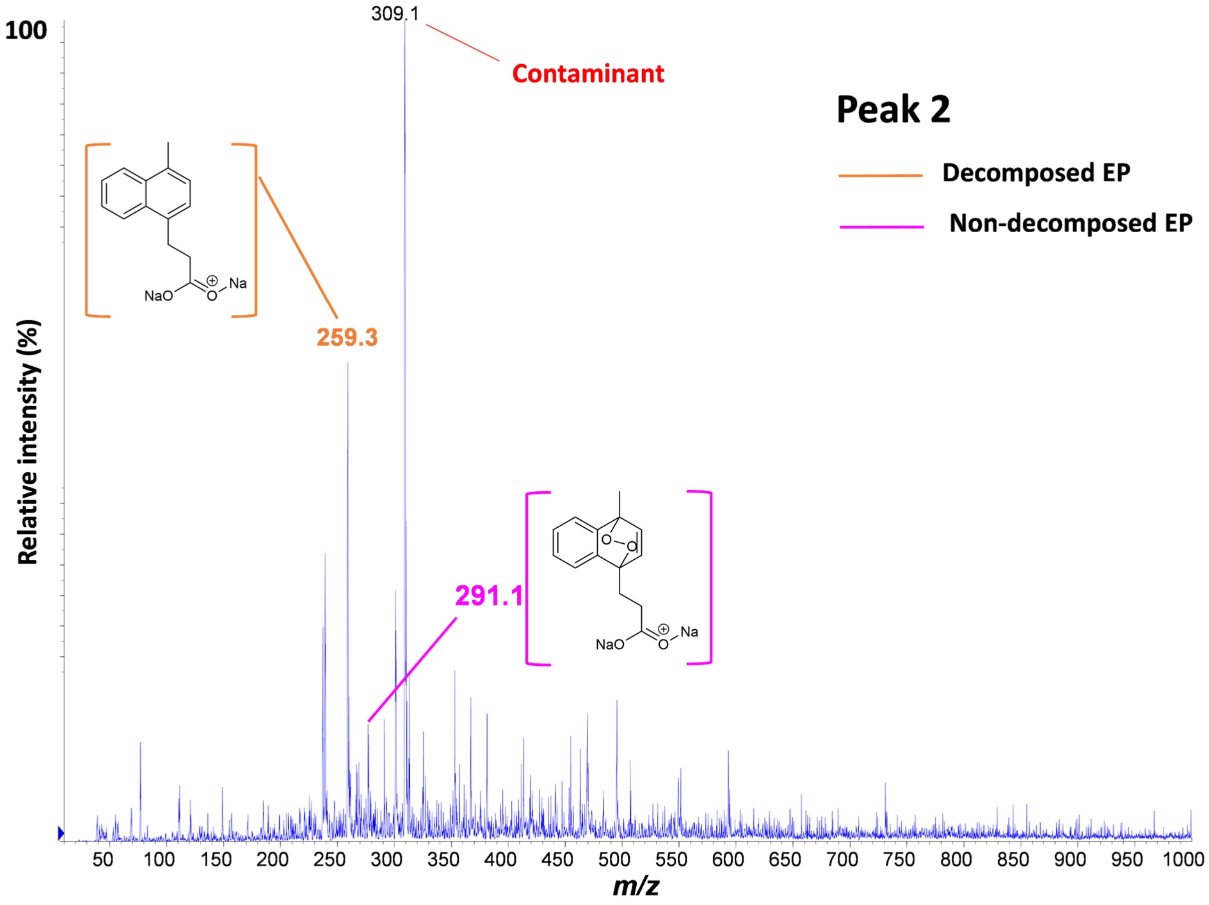

Fig. S6. Q1 scan of the "Peak 2" indicated in the LC-UV chromatograms of EP (Fig. S4).

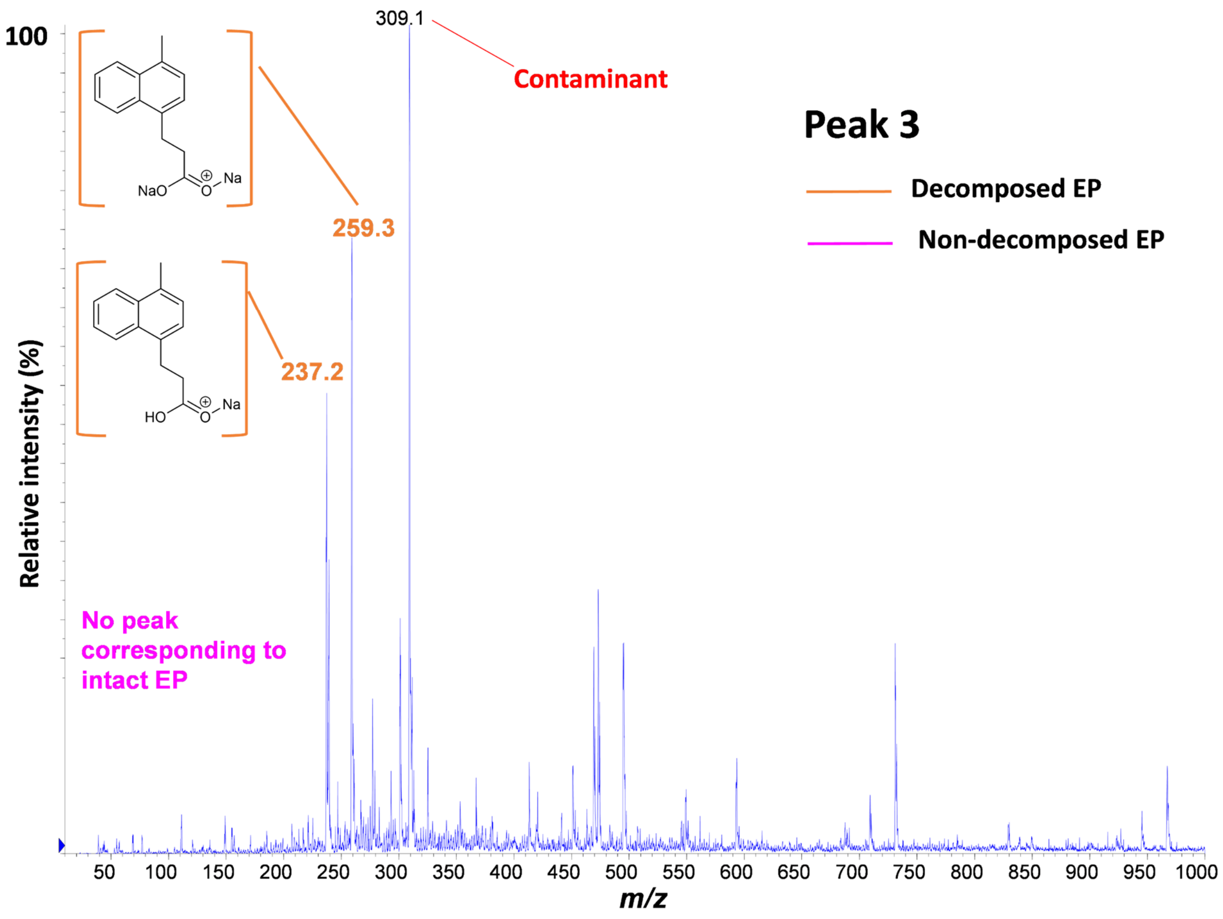

Fig. S7. Q1 scan of the “Peak 3” indicated in the LC-UV chromatograms of EP (Fig. S4).

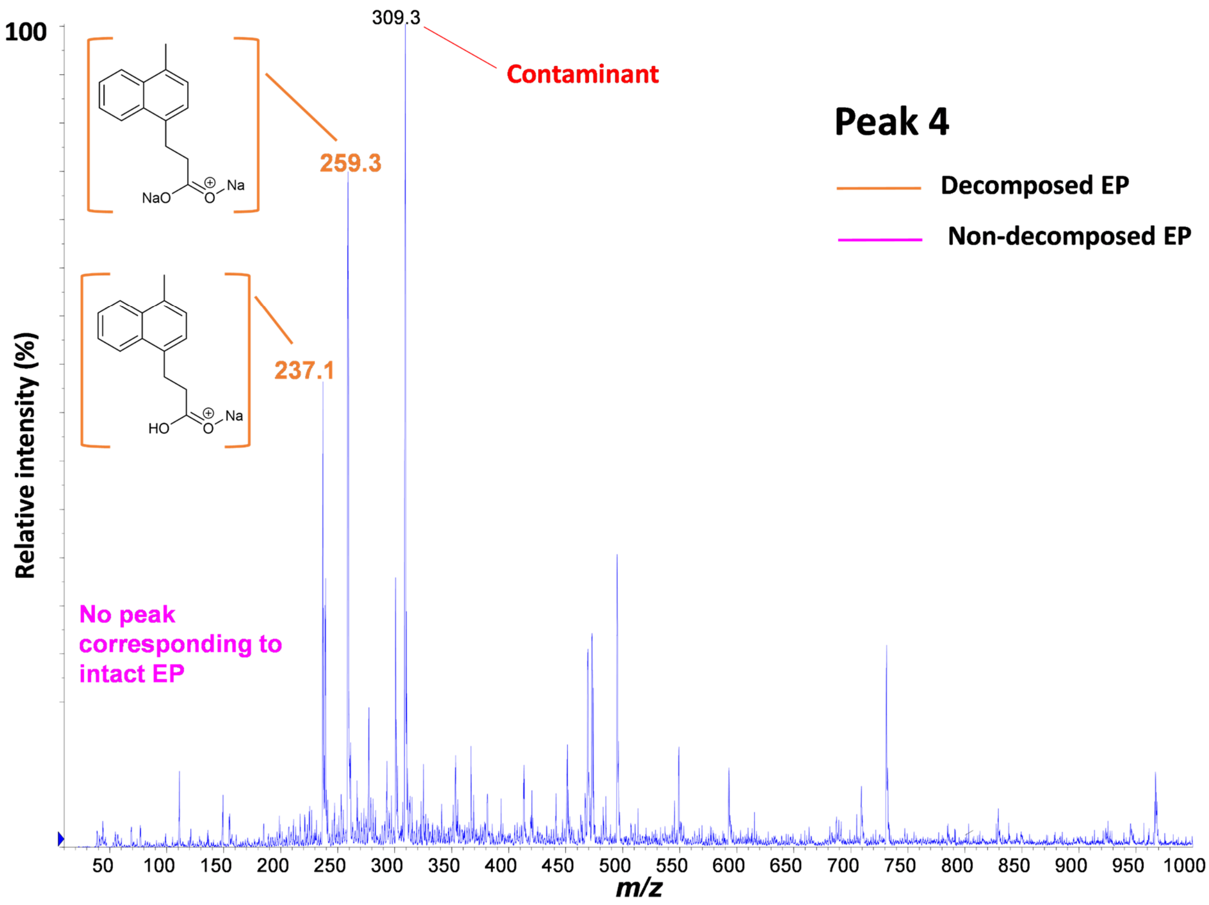

Fig. S8. Q1 scan of the "Peak 4" indicated in the LC-UV chromatograms of EP (Fig. S4).

6- ^{1}H NMR analyses of EP’s decomposition in the absence and presence of SQ-OOHs.

Compatibility between the characteristic peaks of fully decomposed EP can be observed in EP incubated at 25 °C for 18 h, and in EP + SQ-OOHs under the same conditions. Details are presented in the assignments table and spectra below.

Table S1. Characteristic ^{1}H NMR peaks of intact EP (EP 0 h) and decomposed EP (EP 18 h, and EP+SQ-OOHs 18 h).

^{1}H NMR ($CDCl_3$, 400 M Hz), δ (ppm)			
**Position**	EP 0 h	EP 18 h	EP+SQ-OOHs 18 h
**1**	7.26-7.33 (m, 4H)	7.52-7.54 (m, 2H)	7.44-7.47 (m, 2H)
**2**			
**3**		8.01-8.04 (m, 2H)	7.93-7.99 (m, 2H)
**6**			
**8**	6.76 (s, 2H)	7.23 (d, *J* = 7.2 Hz, 2H)	7.16 (d, *J* = 7.2 Hz, 2H)
**9**		7.26 (d, *J* = 6.8 Hz, 2H)	7.19 (d, *J* = 7.2 Hz, 2H)
**11**	2.18 (s, 3H)	2.66 (s, 3H)	2.59 (s, 3H)
**12**	2.70-2.72 (m, 2H)	3.39 (t, *J* = 8.2 Hz, 2H)	3.32 (t, *J* = 8.0 Hz, 2H)
**13**	2.75-2.82 (m, 2H)	2.77 (t, *J* = 8.0 Hz, 2H)	2.68 (t, *J* = 8.0 Hz, 2H)

N/A to the positions: 4, 5, 7, and 10.

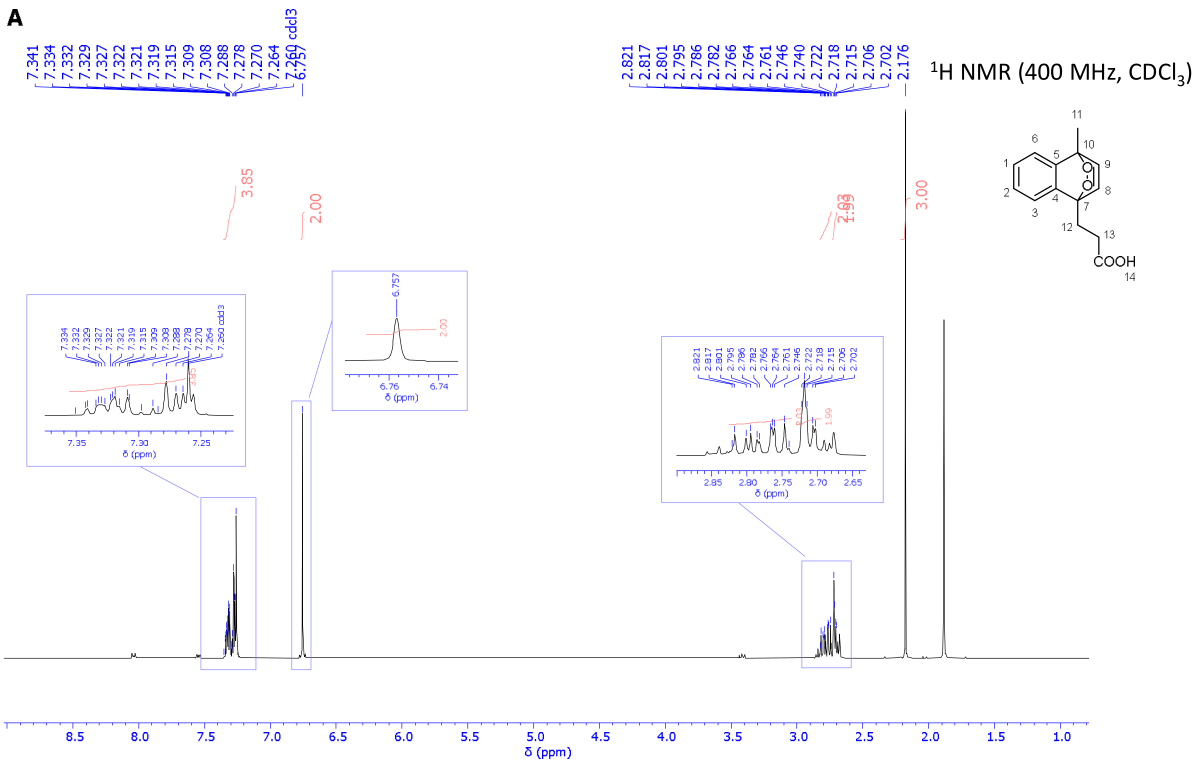

Fig. S9. ^{1}H NMR spectrum of EP at 0 h prior to the start of the incubation.

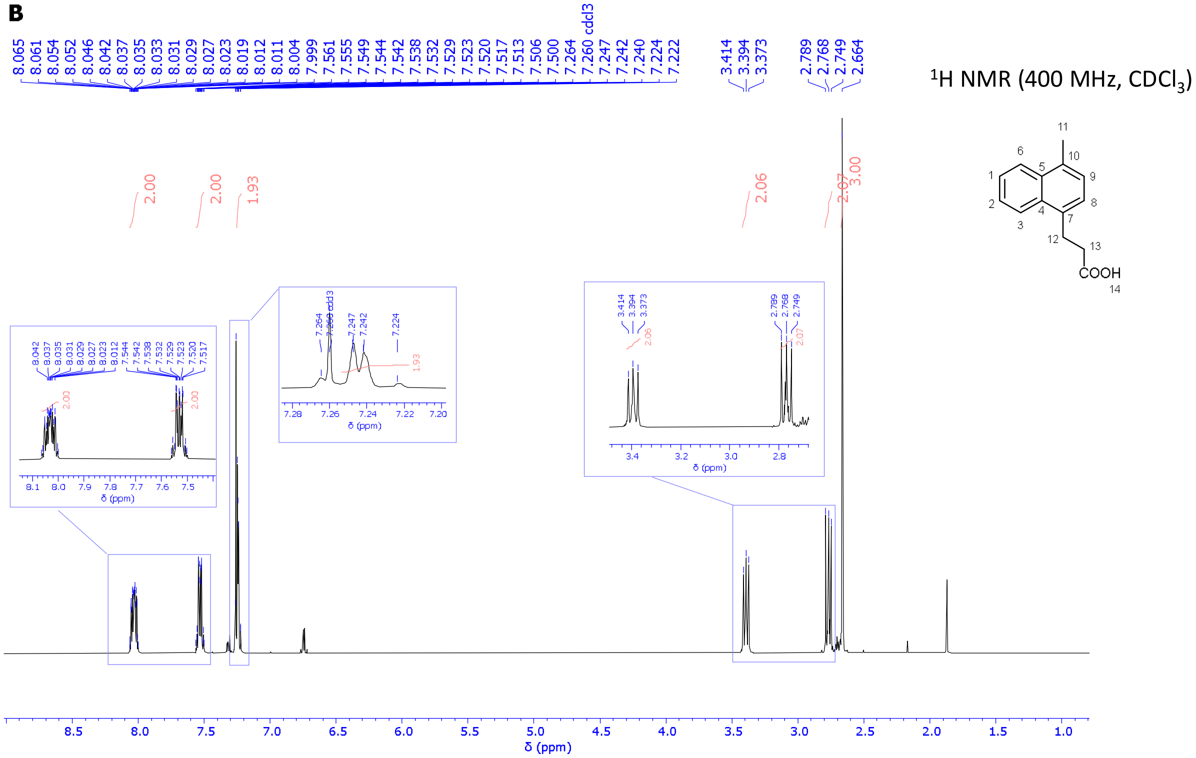

Fig. S10. ^{1}H NMR spectrum of EP decomposition after incubation at 25 °C for 18 h.

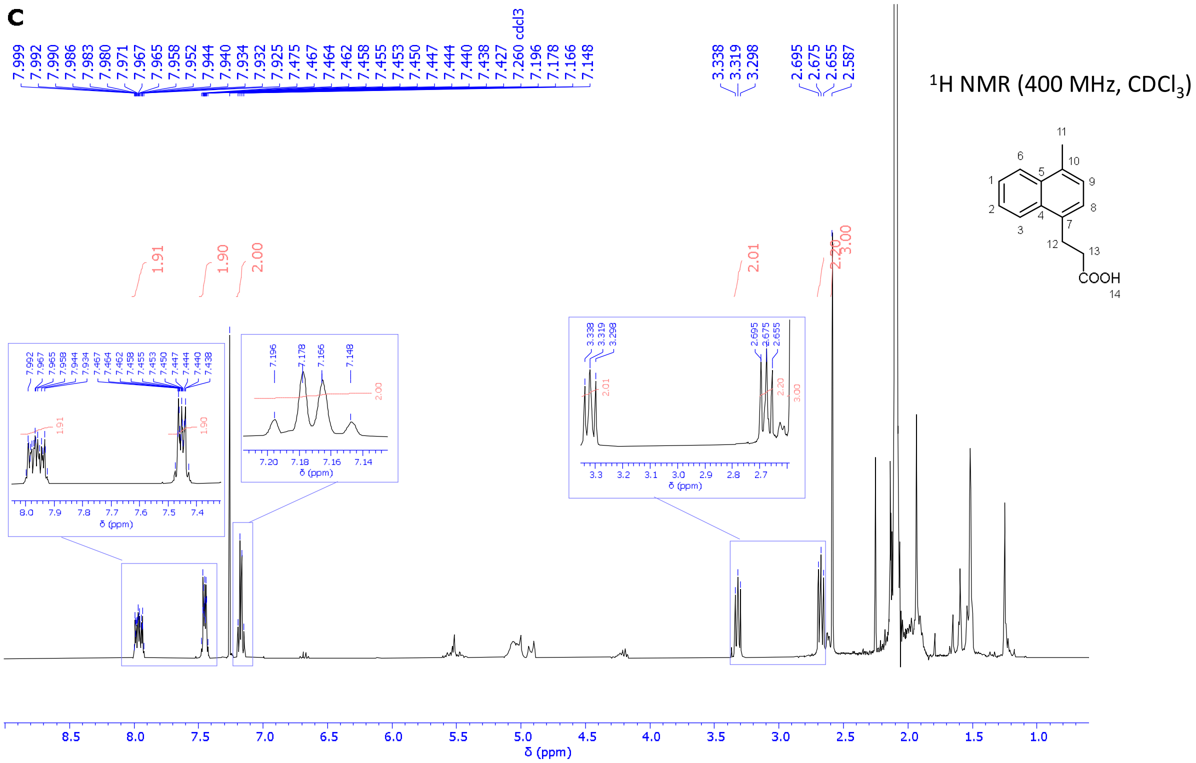

Fig. S11. ^{1}H NMR spectrum of EP+SQ-OOHs decomposition after incubation at 25 °C for 18 h.

7- Proposed EP decomposition mechanisms in the absence and presence of SQ-OOHs.

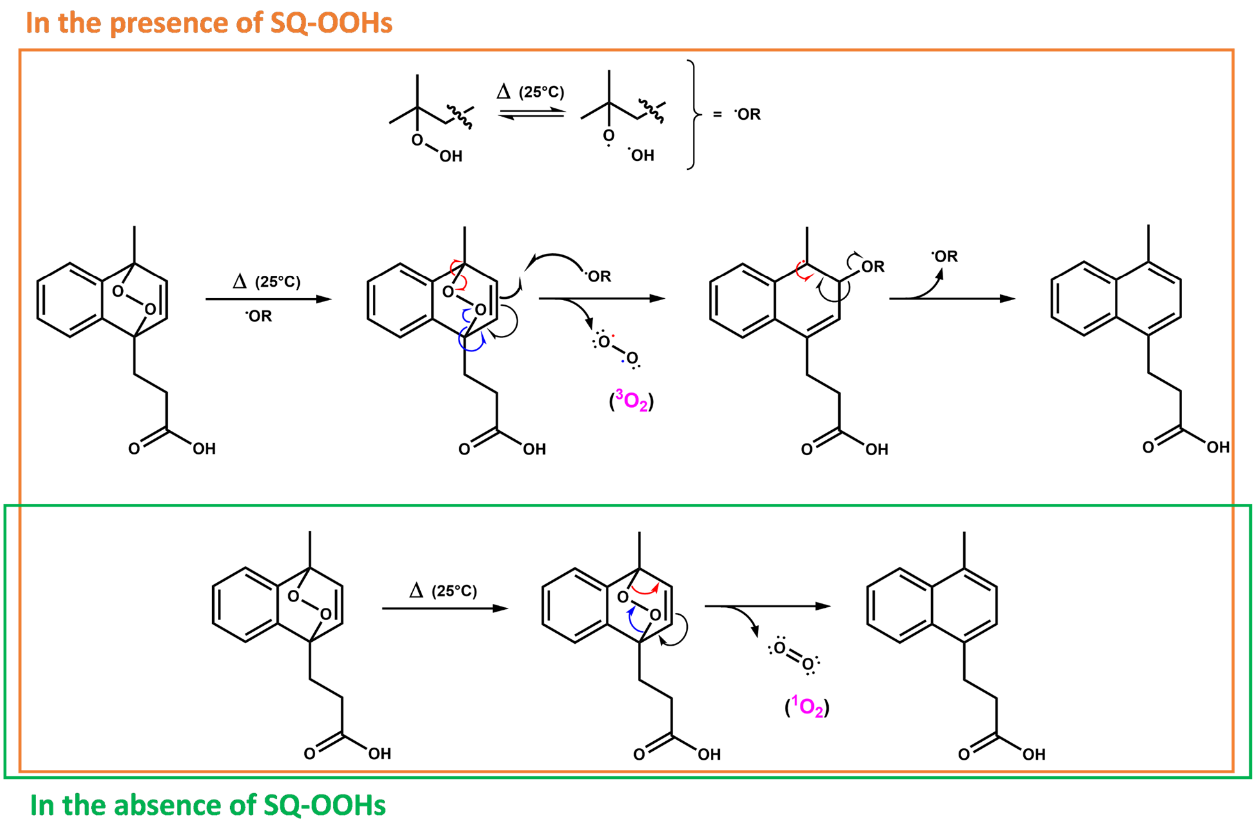

Fig. S12. Expected decomposition mechanisms of EP and proposed radical-induced decomposition of EP promoted by the presence of trace amounts of alkoxyl and hydroxy radicals resulting from SQ-OOHs' O-O homolytic decomposition upon incubation at 25°C.

8- Q1 MS and LC-MS analyses of SQ and SQ-OOHs products upon exposure to ozone.

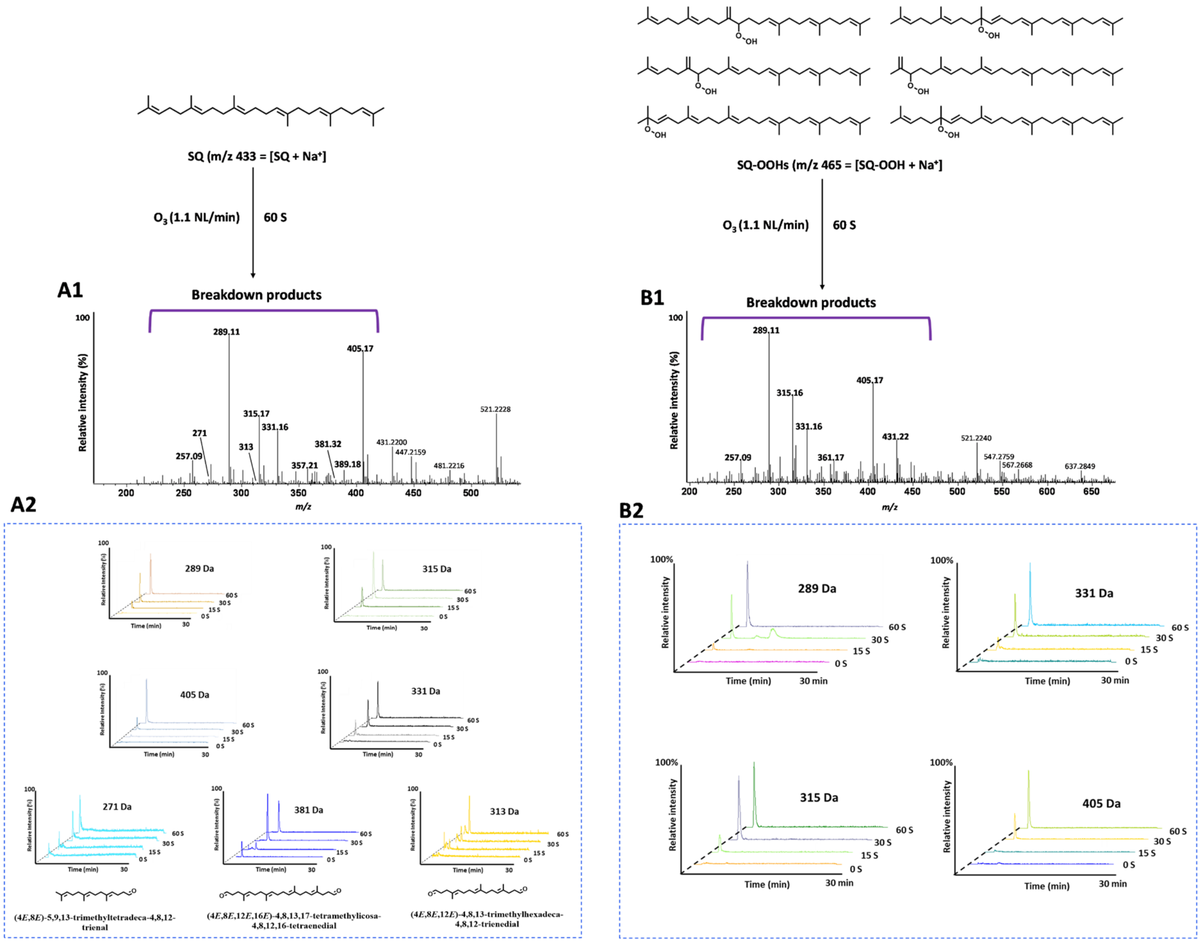

Fig. S13. Q1 MS scan of (A1) SQ ozonolysis and (B1) SQ-OOHs ozonolysis for a period of 60 S. LC-MS chromatograms of the main products observed from (A2) SQ ozonolysis where only 3 species could be identified as spcified in the figure, and (B2) SQ-OOHs ozonolysis for the same period of time. Overall, it can be seen that in both cases, breakdown products were the main produts resulting from the exposure of SQ and SQ-OOHs to ozone.

9- Interpretation of DMPO/tBuOOH ESR spectrum upon UV irradiation.

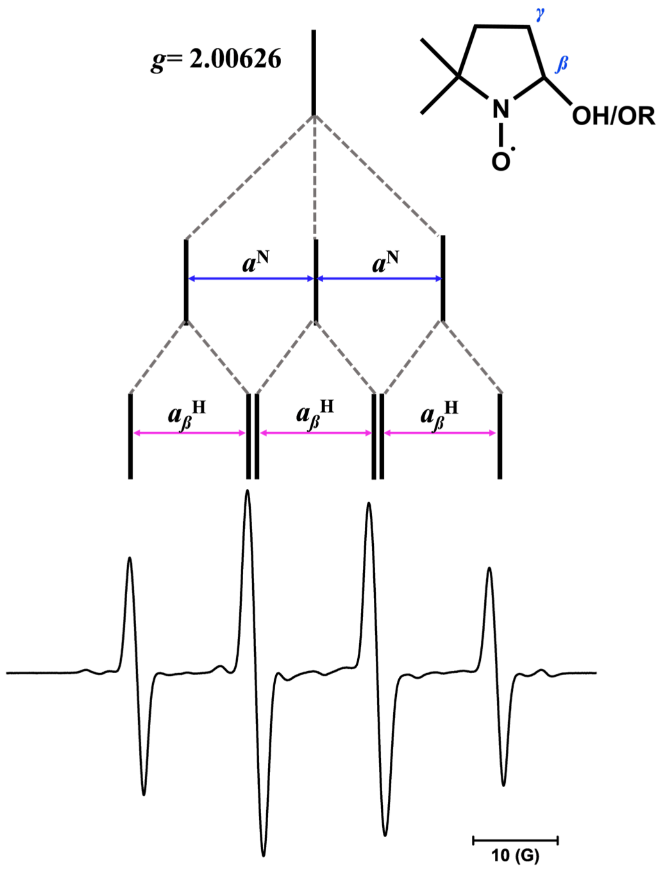

Fig. S14. Stick diagram interpretation of the hyperfine splitting of DMPO-OR generated from the UV irradiation of tBuOOH (a^N=14.29 G and a_β^H≈14.21 G).

10- Interpretation of DMPO/H_2O_2 ESR spectrum upon UV irradiation.

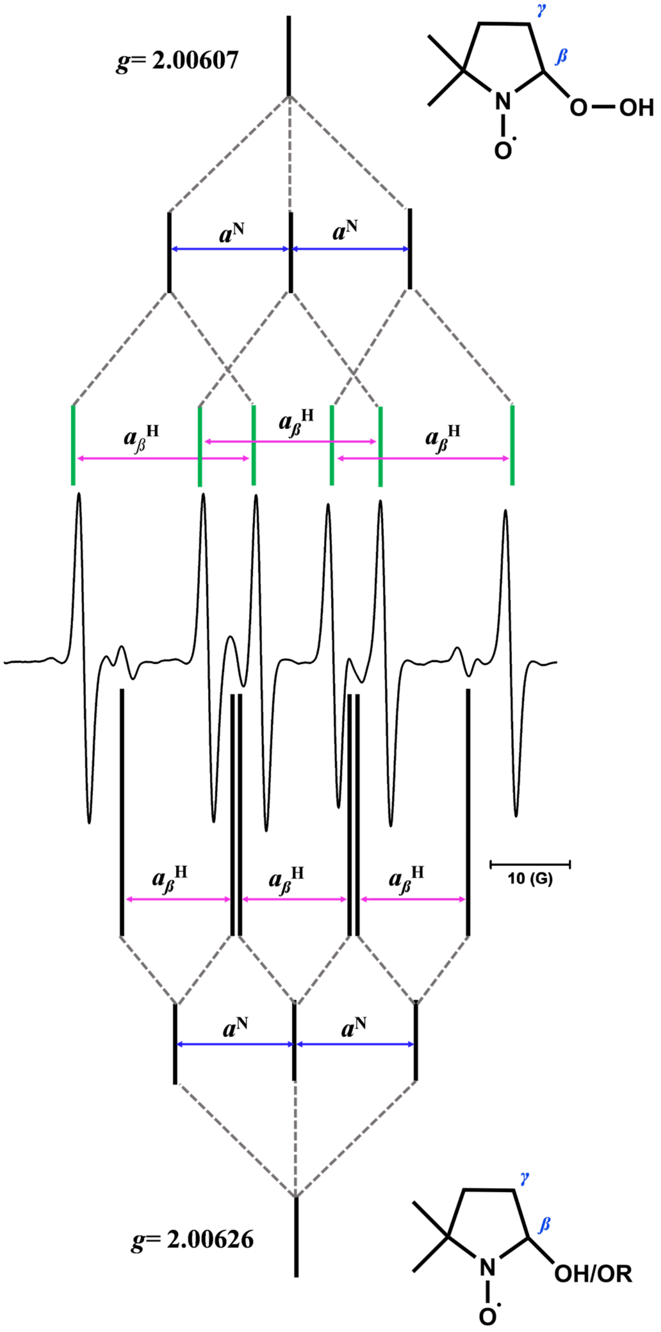

Fig. S15. Stick diagram interpretation of the hyperfine splitting of DMPO-OOH generated from the UV irradiation of H_2O_2 as a major radical (a^N=15.75 G and a_β^H=22.41 G), and of DMPO-OH as a minor radical (a^N=14.23 G and a_β^H≈14.20 G).

11- Interpretation of DMPO/ $O_2^{\bullet-}$ spectrum obtained from KO_2.

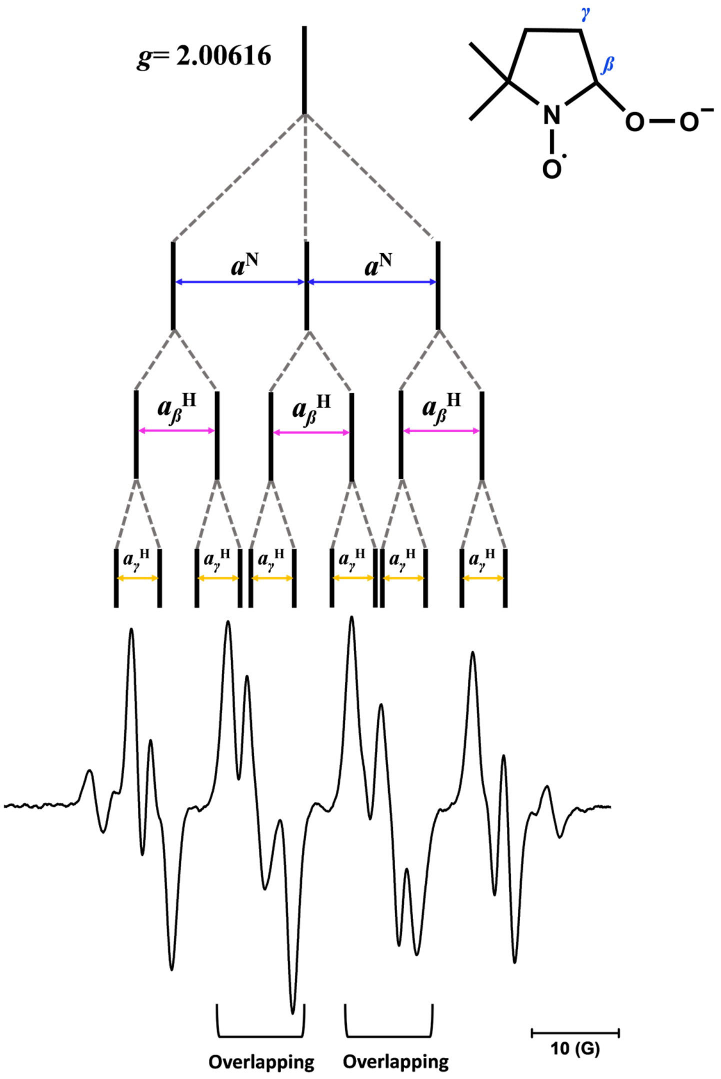

Fig. S16. Stick diagram interpretation of the hyperfine splitting of DMPO-OO⁻ generated from the quenching of potassium from KO_2.

12- Side profile representation of POBN/SQ-OOHs photooxidation.

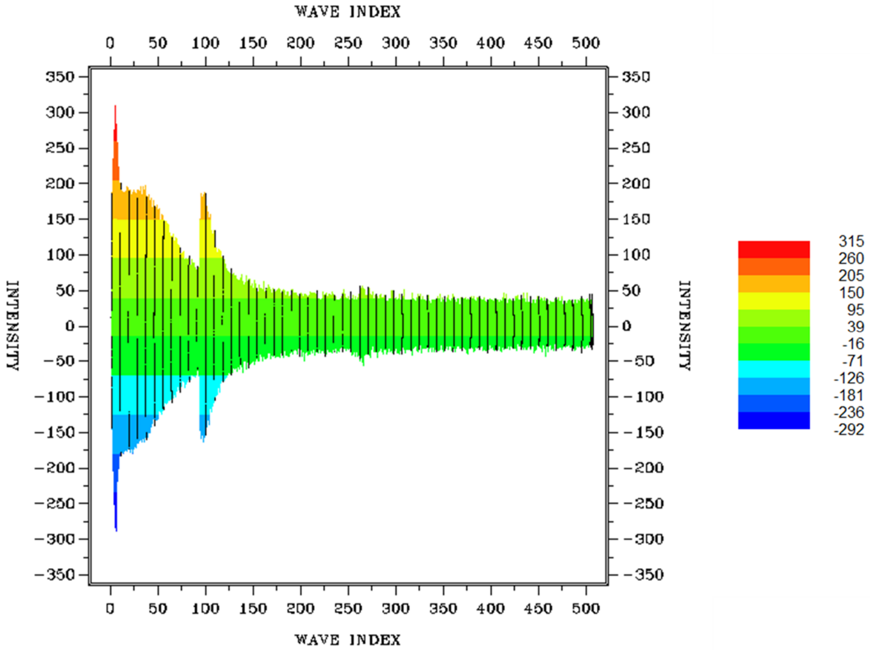

Fig. S17. Side profile representation of the POBN-radical adducts generated throughout the oxidation of SQ-OOHs for 13 h and 30 min. Radicals were mostly observed between 0 h and 4 h (1 wave index = 1.6 min).

13- ESR spectra comparison of DMPO/SQ-OOHs/SOD photooxidation over time with DMPO/SQ-OOHs main spectrum and DMPO/H_2O_2.

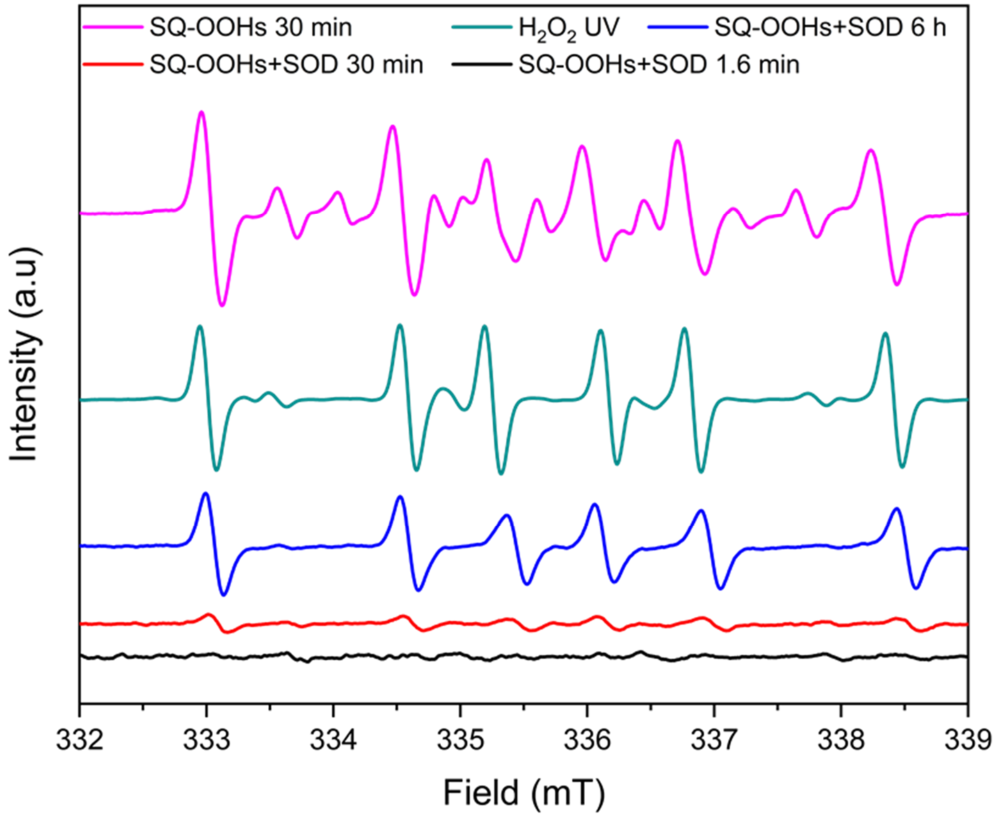

Fig. S18. Comparison of the ESR spctra generated from the photooxidation of DMPO/SQ-OOHs/SOD for 1.6 min, 30 min and 6 h, to spectra obtained from DMPO/SQ-OOHs photooxidation after 30 min, and to DMPO-OOH spectra obtained from the UV irradiation of H_2O_2.

14- LC-MS/MS analysis of 2-OOH-3-(1,2-dioxane)-SQ generated from the photooxidation of SQ-OOHs in the presence of SOD.

To 5 ml of 1.36 mM pure SQ-OOHs in hexane was added 1 ml of 1.1 mg/ml SOD solution in PBS (pH = 7.4), the solution was constantly well stirred to give a homogenous emulsion throughout the photooxidation reaction (60 Klux LED, 25°C). Sampling of triplicates was conducted at 0 h, 2 h, 4 h, and 14 h.

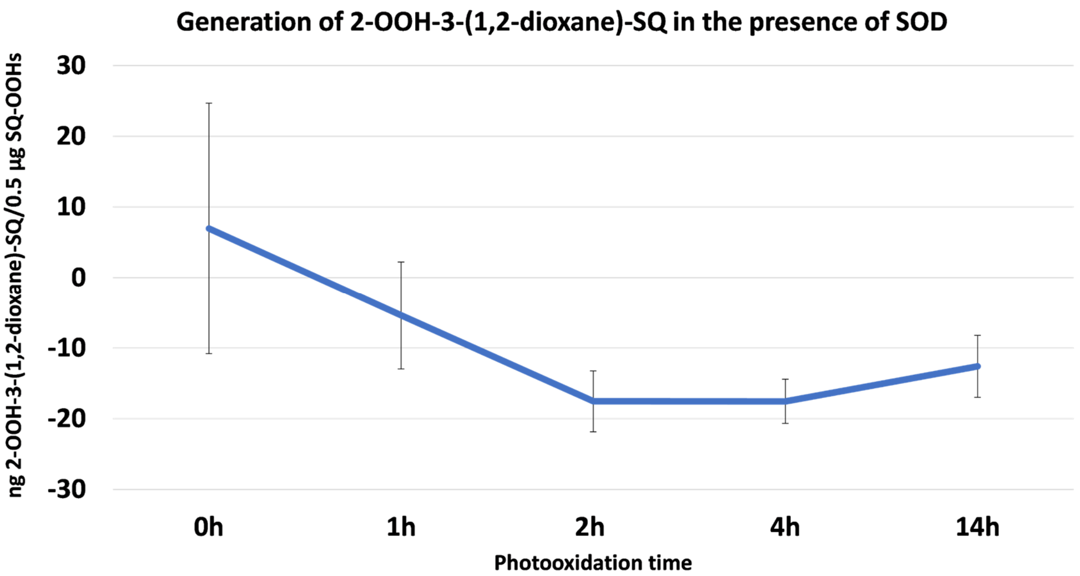

Fig. S19. LC-MS/MS (6500Qtrap (SCIEX, Tokyo, Japan)) analysis of the generated 2-OOH-3-(1,2-dioxane)-SQ from SQ-OOHs photooxidation upon addition of SOD. In this case, the mixture of SQ-OOHs contained a low starting trace amount of 2-OOH-3-(1,2-dioxane)-SQ. Overall, compared to what was observed from the photooxidation of SQ-OOHs in Fig. 3 of the main manuscript, the addition of SOD inhibited the generation of 2-OOH-3-(1,2-dioxane)-SQ. Data expressed as mean ± SD, n=3. (Values expressed in minus fell outside of the quantification range and are considered non-significant.)

Table S 2. 6500QTRAP MRM conditions for the method optimized for 2-OOH-3-(1,2-dioxane)-SQ.

	**MRM conditions**
**Source**	ESI
**Ion polarity**	Positive
**Declustering potential (V)**	
**Entrance potential (V)**	75
**Temperature (°C)**	10
**Ion spray voltage (V)**	
**Curtain gas (psi)**	600
**Collision-activated dissociation gas (psi)**	5500
**Collision energy (V)**	35
**Collision cell exit potential (V)**	
**Ion source gas 1 (psi)**	7
**Ion source gas 2 (psi)**	25

15- Schematic interpretation of the inductive effect's role in the targeting of tertiary SQ-OOHs compared to secondary SQ-OOHs.

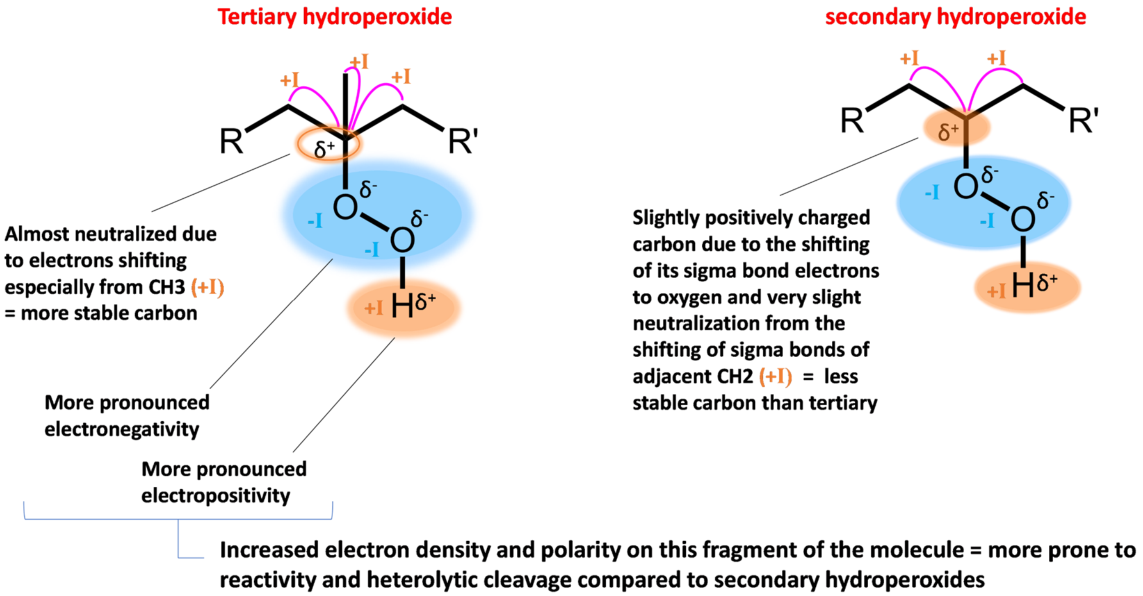

Fig. S20. Schematic representation of the targeting of tertiary SQ-OOHs compared to secondary SQ-OOHs by the described mechanism. The scheme shows the impact of the inductive effect, which was described as an influential factor together with hyperconjugation and the steric effect in the stability of the resulting radicals.

16- LC-UV analysis of the solvent effect on the photooxidation of SQ-OOHs and the generation of 2-OOH-3-(1,2-dioxane)-SQ.

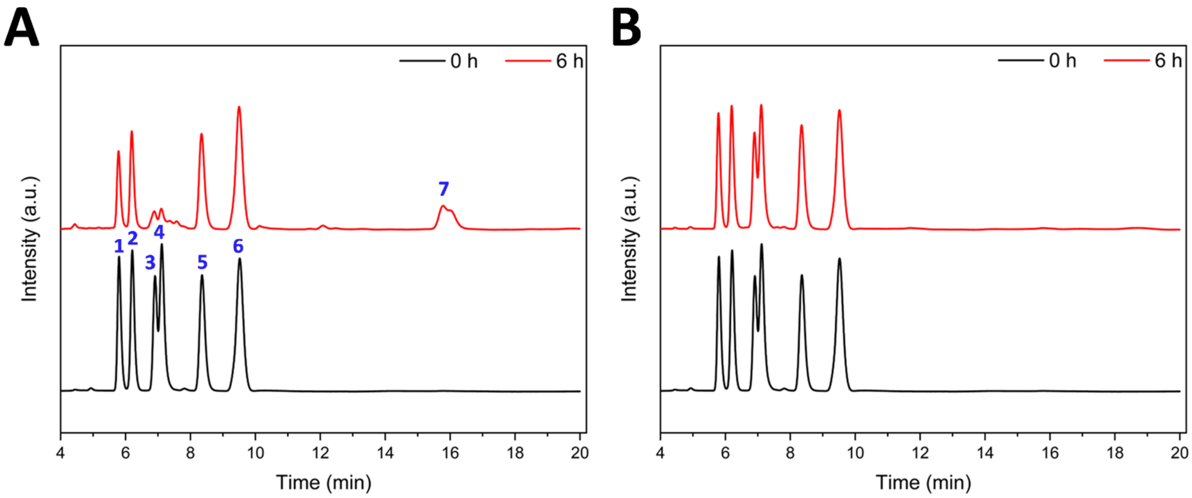

Fig. S21. LC-UV analysis of SQ-OOHs oxidation in (A) hexane and (B) methanol. The peaks numbered from 1-7 are identified respectively as follows: 11-OOH-SQ, 7-OOH-SQ, 10-OOH-SQ, 6-OOH-SQ, 3-OOH-SQ, 2-OOH-SQ and 2-OOH-3-(1,2-dioxane)-SQ. In hexane, an apparent decrease in the isomers 10-OOH-SQ and 6-OOH-SQ was observed with the generation of 2-OOH-3-(1,2-dioxane)-SQ. While in methanol, SQ-OOHs barely decreased over 6 h of photooxidation.

17- Q1 MS analysis of the DMPO adducts formed during SQ-OOHs' photooxidation and thermal oxidation.

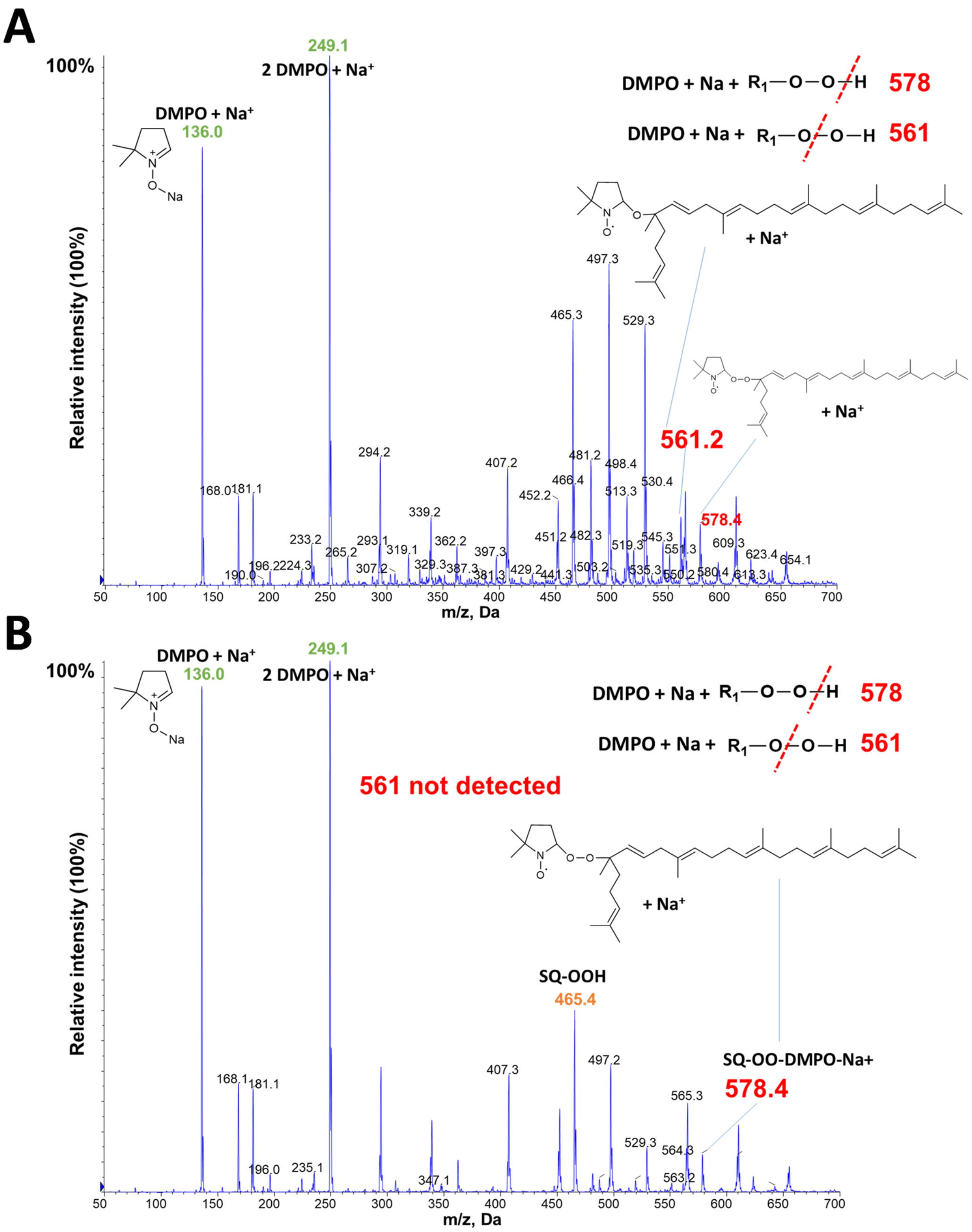

Fig. S22. DMPO radical adducts resulting from SQ-OOHs (A) thermal oxidation, and (B) photooxidation. Under thermal oxidation, a significant number of breakdown species could be observed with the formation of both DMPO-O-SQ and DMPO-OO-SQ, while under photooxidation, there were less breakdown products with one main adduct detected belonging to DMPO-OO-SQ. These observations confirm our proposed hypothesis that under photooxidation, SQ-OOHs' O-H bond is mainly targetted, while under thermal conditions, both O-O and O-H can be cleaved, with the former giving rise to mainly breakdown products.
